# Low light reduces saffron corm yield by inhibiting starch synthesis

**DOI:** 10.3389/fpls.2025.1544054

**Published:** 2025-01-31

**Authors:** Weijing Yang, Xin Li, Fei Chang, Xue Qiu, Xulong Huang, Zhan Feng, Jie Yan, Qinghua Wu, Feiyan Wen, Jin Pei, Tao Zhou

**Affiliations:** ^1^ State Key Laboratory of Southwestern Chinese Medicine Resources, Chengdu University of Traditional Chinese Medicine, Chengdu, Sichuan, China; ^2^ College of Pharmacy, Chengdu University of Traditional Chinese Medicine, Chengdu, Sichuan, China; ^3^ Department of Pharmacy, Zigong Hospital of Traditional Chinese Medicine, Zigong, Sichuan, China

**Keywords:** saffron, low light, corm yield, sucrose, starch, source-sink relationship

## Abstract

The mechanisms by which low light modulates source-sink dynamics, affecting starch synthesis and formation of underground storage organs in geophyte, remain unclear. In this study, a two-year field experiment was conducted under natural light (NL) and low light (LL, 50% of NL intensity) conditions. LL resulted in a 23.66% and 21.23% reduction in corm yield in 2023 and 2024, respectively. Saffron plants under LL had larger, longer leaves with a higher proportion of dry weight (DW) compared to those under NL. Despite the marked inhibition of photosynthetic capacity, initial DW, sucrose and glucose concentrations in leaves were comparable to those under NL. Carbohydrate analysis revealed that starch concentration in the mother corms under LL decreased by 18.00% relative to NL, while sucrose and glucose concentrations increased by 28.44% and 68.44%, respectively. At the corm expansion stage, sucrose concentration in leaves and daughter corms under LL conditions was 17.32% and 54.08% higher than under NL, but glucose and starch concentrations in daughter corms were 22.08% and 10.22% lower, respectively. Additionally, the activity of invertase (INV), sucrose synthase in the decomposition direction (SUS) and ADP-glucose pyrophosphorylase (AGPase) in daughter corms were reduced under LL. LL also affected phytohormones concentrations, with increased levels of indole-3-acetic acid (IAA) and gibberellin (GA_1_) in LL leaves and daughter corms, and decreased abscisic acid (ABA) levels. Transcriptome and quantitative PCR analyses showed that LL upregulated the expression of genes involved in glycolysis and the tricarboxylic acid cycle in leaves, while downregulating *CsSUS*, *CsINV1*, Cs*AGPS1*, *CsZEP*, and *CsNCED*, which are key to sucrose hydrolysis, starch synthesis, and ABA biosynthesis. Exogenous GA_3_ application further inhibited SUS, INV and AGPase activities in daughter corms, indicating that high GA concentrations impair carbohydrate metabolism in these organs. In conclusion, LL decreases saffron corm yield by promoting the allocation of reserves from mother corms to leaves at the seedling stage. By the period of the daughter corms enlargement, elevated GA_1_ and IAA levels and reduced ABA concentration promote leaf growth while inhibiting carbohydrate metabolism in daughter corms, thereby reducing sucrose transport from leaves to daughter corms and suppressing corm yield formation.

## Introduction

Efficient biomass allocation between aboveground and belowground organs is essential for the growth and yield formation of geophytes (Rizzalli et al., 2002; Saldaña Villota et al., 2015). Light intensity not only influences biomass distribution but also affects the transport of assimilates between tissues. However, the mechanisms by which light impacts these processes and yield formation in geophytes remain unclear (Smith and Stitt, 2007). This knowledge gap is particularly significant for saffron (*Crocus sativus* L.), a geophyte cultivated for its valuable stigmas. In regions with reduced sunlight, saffron cultivation often leads to corm shrinkage and yield decline.

Saffron reproduces asexually through corms, and its stigmas-often referred to as “plant gold”-are highly valued not only in medicine, for treating cardiovascular, digestive, and neurodegenerative diseases (Tavakkol-Afshari et al., 2008; Finley and Gao, 2017), but also in dyeing, gastronomy, and perfumery (Mzabri et al., 2019). Iran is the primary center for saffron cultivation, with the majority of production exported to European and Asian markets (Gresta et al., 2009; Shokrpour, 2019; Cardone et al., 2021). In recent years, South and East Asia, particularly China and Japan, have increased saffron cultivation to meet rising market demand (Wang et al., 2021; Kumar et al., 2022). Compared to traditional cultivation areas, these emerging regions experience significantly fewer annual sunshine hours, resulting in insufficient solar radiation and frequent corm shrinkage. Increasing atmospheric aerosol concentrations due to industrial activities have further diminished solar radiation (Khodakarami and Ghobadi, 2016). This trend, especially in developing regions, is expected to continue (Liang and Xia, 2005; Yang et al., 2016). In China, reduced light has already decreased maize (*Zea mays* L.) biomass by 19.7% and yield by 20.6%, while wheat (*Triticum aestivum* L.) yield has decreased by 5.2% (Chen et al., 2013; Zhao et al., 2020). Understanding how low light affects crop growth and yield, as well as developing improved varieties and cultivation strategies, is essential for mitigating these effects.

Under low light, source (energy producing or supplying organs) capacity often fails to meet sink (energy acquiring or consuming organs) demand, reducing crop yields, as seen with saffron (Renau-Morata et al., 2012; Smith et al., 2018; Paradiso et al., 2019). Furthermore, the transport of photosynthates between organs is disrupted, further reducing yield. During seedling establishment, low light increases the preferential allocation of carbohydrates to stems and leaves, promoting aboveground growth to enhance light capture (Kitajima, 1994; Poorter et al., 2012; Perrin and Mitchell, 2013). Several studies have shown that, under low light, soluble sugar concentrations in stems or stem-like organs increase significantly (Koch, 1996; van Doorn, 2008). This preferential allocation of carbohydrates to aboveground parts reduce the energy available for other organs, causing uneven growth. In geophytes like saffron, corms act as energy storage organs, providing the initial energy for leaf development and early daughter corm formation (Sheikh et al., 2022). Field studies have shown that the size of the mother corm is proportional to morphological indicators of growth, such as leaf length, width, and thickness (Cardone et al., 2021). The mother corm contributes 10-20% of the total biomass to daughter corms (Lundmark et al., 2009; Renau-Morata et al., 2012; Ahrazem et al., 2015). Consequently, low light may disrupt carbohydrate distribution in the mother corm, affecting daughter corm yield formation.

Carbohydrate distribution between organs is influenced by the source-sink relationship, organ energy demand, and surplus carbon release (Smith and Stitt, 2007; Smith et al., 2018). Low light reduces the activity of energy metabolism enzymes in sink organs, decreasing energy demand and reducing carbohydrate allocation. The impact of low light on carbohydrate distribution varies by species and growth stage. For example, in lentil (*Lens culinaris* L.), low light increases carbohydrate allocation to roots to enhance nutrient uptake (Vollsnes et al., 2012). In rice (*Oryza sativa* L.), low light increases carbohydrate retention in leaves to sustain photosynthesis (Xu et al., 2006). In wheat, low light increases carbohydrate allocation to shoots during the vegetative stage but shifts allocation to grains during the reproductive stage to ensure successful reproduction (Dong et al., 2014; Olszewski et al., 2014). In Iran, saffron leaf expansion begins after transplanting and continues before the rapid enlargement phase of daughter corms, with 60 days overlap between leaf and daughter corm enlargement (Fallahi and Mahmoodi, 2018). Once leaf DW and length stabilize, biomass shifts from leaves to the daughter corm, which then serve as the primary sink organ, continuing to expand through photosynthesis and remobilized photo-assimilates (Behdani and Fallahi, 2015; Fallahi and Mahmoodi, 2018). Plants under low light condition may alter the extension of the leaf expansion period (Kikuzawa, 1995; Franklin, 2008; Xu et al., 2021a). Studying carbohydrate distribution patterns between leaves and daughter corms helps us to understand the mechanisms by which low light affects daughter corm yield formation.

The enlargement of underground stems is typically accompanied by starch accumulation (Yu et al., 2015; Dong et al., 2021; Zierer et al., 2021). Starch synthesis is influenced by substrate concentration and the activity of related metabolic enzymes. Sucrose concentration and the catalytic efficiency of starch-synthesizing enzymes significantly impact starch synthesis and underground stem development (Hawker et al., 1979; Sharma and Deswal, 2021; Fang et al., 2022). In saffron corms, starch accumulation correlated positively with the activity of AGPase and SUS, and the trends of the three over time were consistent (Pallotti et al., 2024). In rice, low light reduces AGPase and SUS activities by 39.52% and 20.62%, respectively, resulting in a 39.4% reduction in grain weight (Li et al., 2022). The expression of AGPase and SUS genes is light-dependent, low light downregulating these genes and reducing enzyme synthesis (Gregoriou et al., 2007; Li et al., 2022). Low light may also indirectly influence enzyme activity by altering endogenous phytohormone concentrations. In *Lycoris radiata* and gladiolus (*Gladiolus hybridus*), GA_3_ inhibits SUS and AGPase activities by downregulating genes expression, adversely affecting starch accumulation and bulb development. In contrast, ABA promotes starch accumulation (Li et al., 2021; Xu et al., 2021b). Under short-term shading, plants increase GA concentration to enhance shade avoidance responses (SAR), but prolonged shading typically reduces GA concentrations in aboveground tissues. For example, after 15 days of shading, soybean (*Glycine max* (L.) Merr.) seedlings exhibited a 57.8% reduction in GA_1_ concentration in stems compared to those under natural light (Yang et al., 2018). However, in underground organs, GAs concentration may remain higher under long-term shading conditions than under natural light. Research on sweet potatoes (*Ipomoea batatas* (L.) lam) showed that root GA_3_ concentration was significantly higher than under natural light, while GA_3_ concentrations in leaves were lower (Wang et al., 2014).

The primary objective of this study was to examine carbohydrate distribution patterns between mother corms, leaves, and daughter corms under low light conditions, and their impact on daughter corm yield formation. The field experiments were conducted from 2022 to 2024 with two treatments: NL and LL. A comprehensive analysis of morphological, physiological, and biochemical aspects was performed from the seedling stage to the enlargement of the daughter corm. We hypothesized that low light: 1) promotes the allocation of stored carbohydrates from mother corms to leaves, thereby reducing carbohydrate flow to daughter corms; 2) extends the leaf growth period, retaining more carbohydrates in leaves and reducing sink strength in daughter corms by suppressing starch synthesis enzyme activity. These factors likely inhibit carbohydrate transport from leaves to corms, reducing daughter corm yield.

## Materials and methods

### Plant materials

The corms used in this study were sourced from Jiande, Zhejiang, one of China major saffron production bases. To ensure plant uniformity, corms weighing 20~25 g plant^-1^ were selected for the experiment.

### Site description

Two field experiments were conducted from November 2022 to April 2023 in Pengzhou City (31.19°60’N, 103.75°00’E) and from December 2023 to April 2024 in Chongzhou City (30.56°60’N, 103.64°00’E), both located in Sichuan, China. The soil at both sites is classified as Ochric Aquic Cambosol. Although the climatic conditions were similar at the two locations, Pengzhou experienced lower total solar radiation and average temperatures compared to Chongzhou. The daily sunshine duration, average air temperature, and precipitation during the plant growth period are provided in **Supplementary Figure S1**.

The chemical properties of soil in 0~20 cm layer at Chongzhou site were as follows: pH (1:2.5, soil: water) 6.4, organic matter 20.3 g kg^-1^, total N 2.0 g kg^-1^, available N 125 mg kg^-1^, Olsen-P 17.0 mg kg^-1^, exchangeable K 124 mg kg^-1^, and cation exchange capacity 22.4 cmol kg^-1^. In Pengzhou site, the corresponding values were: pH (1:2.5, soil: water) 6.7, organic matter 23.1 g kg^-1^, total N 2.2 g kg^-1^, available N 125 mg kg^-1^, Olsen-P 16.0 mg kg^-1^, exchangeable K 110 mg kg^-1^, and cation exchange capacity 22.0 cmol kg^-1^.

### Experimental design

The field experiment was arranged in a randomized block design with three replicates. Two light intensity treatments were applied: NL and LL. Previous studies indicated that saffron exhibits a strong SAR when grown under light intensity below 50% of NL in this region, which led to the establishment of the 50% NL intensity treatment using shade cloth. The shade cloth was supported by a stainless-steel framework (20×5×2 m^3^). Temperature sensors, positioned 30 cm above the ground, monitored the temperature inside the shaded area, and if a temperature difference between inside and outside was detected, a fan was used to ensure proper airflow and cooling. Each plot measured 1.5×5 m^2^, with a 0.5 m buffer between plots. Row spacing was 15 cm, and the plant density was 45 corms m^-2^. Pre-sowing basal fertilizers included 50 kg N ha^−1^ (urea), 160 kg P_2_O_5_ ha^−1^ (triple superphosphate), and 100 kg K_2_O ha^−1^ (potassium chloride). Additional nitrogen was applied with irrigation at rates of 50 kg N ha^−1^ in December and January, and 100 kg N ha^−1^ in February. All plots were manually weeded monthly and irrigated reasonably according to the precipitation of the month during the growth period.

Physiological and biochemical indicators such as plant phenotype, yield, leaf photosynthetic intensity, carbohydrate concentration and related metabolic enzyme activity, and phytohormone levels were measured. In March 2023, transcriptome analysis was performed on leaf and daughter corm samples to examine gene expression patterns linked to carbohydrate metabolism and hormone regulation.

In March 2024, an exogenous phytohormone application experiment was conducted under the NL treatment to assess the role of GA and the GA to ABA ratio on carbohydrate metabolism enzyme activity in daughter corms. This experiment followed a completely randomized design with four treatments: sterile water (control), 25 μM, 50 μM, and 100 μM gibberellin (GA_3_, Sigma), each with three replicates of 3 corms. The concentrations and methods for each treatment were based on our previous study (Zhou et al., 2024). Each plant was sprayed with 20 mL of GA_3_ solution, with a second application the following day. Fresh daughter corm samples were collected one week later to measure enzyme activity.

### Plant sampling

After completing bud development and flowering indoors, saffron plants were transplanted to the field on 1 November, 2022 and 5 December, 2023. Sample collection began 30 days after transplanting and continued monthly thereafter until March. The plants were divided into leaves, mother corms, and daughter corms, with three plants frozen in liquid nitrogen and stored at -80°C for enzyme activity and phytohormones analysis. Leaves and corms were separated from the rest of the three plants, after measuring the morphology of the leaves, the leaves and corms were dried at 80°C and ground for further carbohydrate concentration analysis. In the experimental area, saffron is cultivated until harvest in April. Corm development begins in January and accelerates rapidly in March; thus, many of our observational measurements were collected during March.

### Determination of leaf morphology

To assess the effect of light intensity on plant growth, leaf morphological was recorded regularly after transplanting the plants into the field. After separating the leaves, their length was measured with a tape measure. A small segment (approximately 5 mm long) was cut from the intersection with the leaf sheath using a sharp surgical knife, immediately stored in FAA fixative solution, and later examined using a T-type microscope to measure thickness and width. Over 30 leaf segments were observed for each replicate. Leaf area was calculated using the following formula (Sepaskhah et al., 2013):


A=0.9×L×W×0.1

(A, leaf area, cm^2^. L, leaf length, cm. W, leaf width, mm. 0.9, leaf area index.)

### Calculation of DW of each organ and yield of daughter corms

After drying the leaves and corms at 80 °C to a constant weight, they were weighed using a balance to obtain DW. At the end of the vegetative growth in April, the daughter corms were dug up and categorized into four grades, ≤ 8.0 g, 8.0-15.0 g, 15.0-25.0 g, and ≥ 25 g (Zhou et al., 2022). The number and weight of daughter corms in each grade were measured, and the total yield was calculated by summing the weights of all four grades. Yield per unit area (g m^2^) was calculated based on a combination of the average weight (g) per corm per plot and the number of plants per unit area (m^2^).

### Determination of leaf photosynthetic parameters and chlorophyll concentration

The net photosynthetic rate of leaves was measured using the Li-6400 photosynthesis system (Li-COR, Lincoln, NE, USA) once a month from December 2022 to March 2023, between 10:00 and 12:00 on sunny days. Three leaves were selected, and measurements were taken about 5 cm above the membranous sheath. The photosynthetic photon flux density was set at 1000 μmol m^−2^ s^−1^, with a CO_2_ flow rate of 440 μmol mol^−1^ and a chamber temperature was 20°C (Zhou et al., 2022). Chlorophyll fluorescence was measured using the MINI-PAM-II Portable Fluorometer (Walz, Effeltrich, Germany) in March 2024, following the procedure described by (Ke et al., 2023).

Photosynthesis light-response curves were measured using the Li-6800 (Li-COR, Lincoln, NE, USA). Measurements were made in the field in March, 2024. The measurements were taken between 10:00 and 12:00 on sunny days, following a 20- min light induction with photosynthetically active radiation (PAR) of 1600 μmol m^−2^ s^−1^ for NL and 1200 μmol m^−2^ s^−1^ for LL conditions (Gao et al., 2022). For each PAR level (ranging from 2400 μmol m^−2^ s^−1^ to 0 μmol m^−2^ s^−1^ for NL and from 1600 μmol m^−2^ s^−1^ to 0 μmol m^−2^ s^−1^ for LL), the measurement time was controlled to 180 s. Light-response parameters were automatically recorded by the instrument, the photosynthetic parameters such as the light compensation point, light saturation point, maximum net photosynthetic rate, dark respiration, and initial quantum efficiency were fitted using a modified right-angle hyperbolic model in photosynthetic version 4.1.1.

Fresh leaf samples were ground in liquid nitrogen, and 0.1 g of powder was extracted in 10 mL of 80% (v/v) aqueous acetone at 25°C in the dark for 48 h. Absorbance was measured using a spectrophotometer (UV-1900, China) at 645 nm, and 663 nm. Chlorophyll concentrations were calculated using the following formulas (Arnon, 1949; Porra et al., 1989):


$$\begin{array}{c} \text{Chlorophyll a }\left(\text{Chl a}\right)\\ =(12.7\times \text{A }663-2.69\times \text{A }645)\times \text{V}{\left(1000\times \text{W}\right)}^{-1} \end{array}$$

$$\begin{array}{c} \text{Chlorophyll b }\left(\text{Chl b}\right)\\ =\left(22.9\times \text{A }645-4.68\times \text{A }645\right)\times \text{V }{\left(1000\times \text{W}\right)}^{-1} \end{array}$$

(V, total volume of extract, mL. W, Leaf fresh weight, g.)

### Determination of enzyme activities associated with carbohydrate metabolism

After grinding the samples in liquid nitrogen, biochemical indicators in leaves and mother corms were measured using assay kits from Solarbio Life Sciences (Beijing, China) including sucrose synthase in the synthesis direction (SS), sucrose phosphate synthase (SPS), Ribulose-1,5-bisphosphate carboxylase/oxygenase (Rubisco) and α-amylase (Chen et al., 2022a; Yu et al., 2022; Liu et al., 2023). The activities of INV, SUS and AGPase in daughter corms were measured using ELISA kits from Fantaibio Sciences (Shanghai, China), following extraction with phosphate buffer (Ge et al., 2024). Enzyme activities were determined using respective assay kits, with spectrophotometric readings taken on a microplate reader (ID 5, Molecular DEVICES, USA).

### Determination of sugars and starch

The dried samples were ground, and 40 mg of powder was placed in 10 mL centrifuge tubes, followed by the addition of 5 mL of 80% (v/v) ethanol. The tubes were incubated in a water bath at 80°C for 10 min and then centrifuged at 4500 r for 10 min at 4°C. This extraction process was repeated twice, and the supernatants were combined. The sucrose and glucose were analyzed using equipment and chromatographic conditions as described in (Zhou et al., 2022).

The residue after sugar extraction was used for starch analysis. The precipitate was washed twice with deionized water and then gelatinized for 10 min at 100 °C. After cooling, 0.5 mL of 9.2 mol L^-1^ perchloric acid was added to extract for 15 min, and the supernatant was collected after centrifuging at 5000 r for 10 min after the volume was fixed to 2 mL in deionized water. The remaining residue was extracted repeated once with 4.6 mol L^-1^ perchloric acid, the combined supernatant was determined using the anthrone method (Setter et al., 1994).

### Observation of starch morphology in corms

Dried corms were cut vertically from the center and broken by hand to obtain natural cross-sections, which were cut into 1 mm thick samples. The samples were placed on copper conductive tape with the cross-section facing up and secured with double-sided adhesive tape. A thin layer of gold was sprayed on the samples using an CSC metal ionizer (Cressington 108AUTO, GBR). The samples were then observed and photographed under a scanning electron microscope (EVO MA/LS, ZEISS, Germany) (Zhou et al., 2022).

### Measurement of ABA, IAA and GA

The fresh samples were ground into powder in liquid nitrogen and then freeze-dried, following the extraction and detection procedure described by (Zhou et al., 2024). ABA, IAA and GA were detected by MetWare (http://www.metware.cn/) using the AB Sciex ATRAP 6500 LC-MS/MS platform.

### RNA isolation, sequencing and data analysis

Saffron leaves and daughter corms were collected, labeled, and stored in a -80°C freezer. Total RNA was extracted using Trizol reagent, and RNA quantity was analyzed using a Bioanalyzer 2100 and RNA 6000 Nano LabChip Kit (Agilent, CA, USA, 5067-1511). RNA libraries were constructed using the Illumina^®^ TruSeq™ RNA Sample Preparation Kit (Vennapusa et al., 2020). All samples were sequenced using the Illumina NovaSeq 6000 sequencer by LC-Bio (Hangzhou, China).

The reads were trimmed using Cutadapt version 1.9 and verified using FastQC version 0.10.1. Clean reads were assembled *de novo* using Trinity version 2.15, and expression level were quantified using Salmon (Grabherr et al., 2011; Patro et al., 2017). Differentially expressed genes (DEGs) were selected based on a log_2_ (fold change) >1 or log_2_ (fold change) <-1 and a *p*-value < 0.05 using the R package edgeR version 3.40.2 (Robinson et al., 2010).

Functional annotation was performed by aligning genes to the non-redundant (Nr) protein database (http://www.ncbi.nlm.nih.gov/), Gene ontology (GO) (http://www.geneontology.org), SwissProt (http://www.expasy.ch/sprot/), Kyoto Encyclopedia of Genes and Genomes (KEGG) (http://www.genome.jp/kegg/) and eggNOG (http://eggnogdb.embl.de/) databases using DIAMOND version 2.0.15 (Buchfink et al., 2015). KEGG pathway enrichment was analyzed using KOBAS software version 2.0 (Xie et al., 2011). Heat maps were generated using the OmicStudio tools at https://www.omicstudio.cn/tool.

Raw Illumina sequence data were deposited in the Short Read Archive of the NCBI database (Biosample accession numbers are from SAMN44442459 to SAMN44442470).

### qRT-PCR validation

RNA from saffron leaves and daughter corms was extracted and reverse transcribed using the PrimeScript™ RT Kit (TaKaRa, Japan). PCR was performed using SYBR Green-based assay on a Real Time PCR System (CFX96TM Optics module, USA). Transcription levels were normalized using *CsACT* as the reference gene (Zhou et al., 2020). Primers used are listed in **Supplementary Table S1**, and gene expression was calculated using the 2^-ΔΔCT^ method (Taski-Ajdukovic et al., 2012).

### Statistical analysis

Data on length, DW, yield, carbohydrate, phytohormone concentration, biochemical indicators, and gene expression level (after calculating TPM-Transcripts Per Kilobase of exon model per Million mapped reads) were determined using Student’s t-tests after homogeneity of variances was assessed with F-tests using SPSS statistical software package (Version 24.0, SPSS Institute Inc., USA). Enzyme activities in daughter corms after exogenous treatments were analyzed using one-way ANOVA, with significant differences separated by the LSD test at the level of *p* ≤ 0.05. The figures were drawn using Origin 2023b (Origin, USA).

## Results

### LL increases leaf DW proportion and reduces corm yield

**Figure 1:**
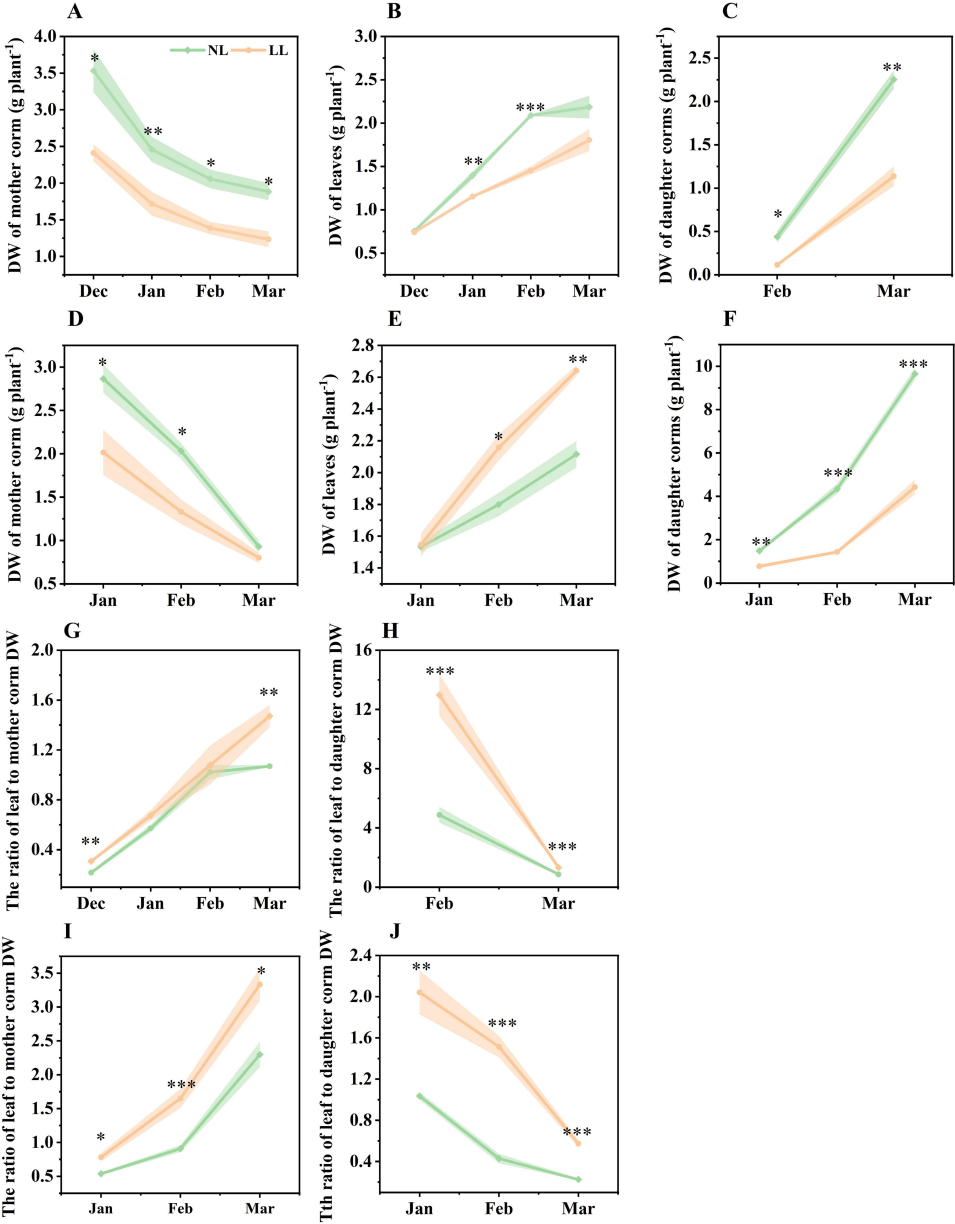
Effect of LL on the dry weight (DW) of organs **(A–F)**, ratio of leaf to mother corm DW **(G, I)** and ratio of leaf to daughter corm DW **(H, J)**. The experiments conducted in 2022~2023 **(A–C, G, H)** and 2023~2024 **(D–F, I, J)**, and the plants were grown under low light intensity (LL) and natural light intensity (NL). Samples were manipulated monthly since December in 2022 and January in 2024. All data were the mean of three replicates. The shadow besides the line in the figure was the range of standard deviation. Significant differences between LL and NL treatments were determined using Student’s t-test, *p ≤ 0.05, **p ≤ 0.01, ***p ≤ 0.001.

From December to March of the following year, the DW of leaves and corms was measured monthly. As the plants developed, the DW of the mother corm steadily decreased, while the DW of leaves and daughter corms progressively increased (**Figure 1**). Under LL conditions, mother corm DW was consistently lower than under NL (**Figures 1A, D**). In December 2022, there was no significant difference in leaf DW between LL and NL conditions. However, after December, the leaf DW under LL conditions was significantly lower than under LL (**Figures 1B, E**). Similarly, the DW of daughter corms was consistently lower under LL conditions compared to NL, starting from their initial formation (**Figures 1C, F**).

The ratio of leaf to mother corm DW continuously increased throughout the growing period, while the ratio of leaf to daughter corm DW decreased. Notably, the proportion of leaf DW relative to both mother and daughter corms was significantly higher under LL compared to NL (**Figures 1G–J**). These findings indicate that under LL, a greater proportion of biomass is allocated to the leaves at the expense of daughter corm development.

**Figure 2:**
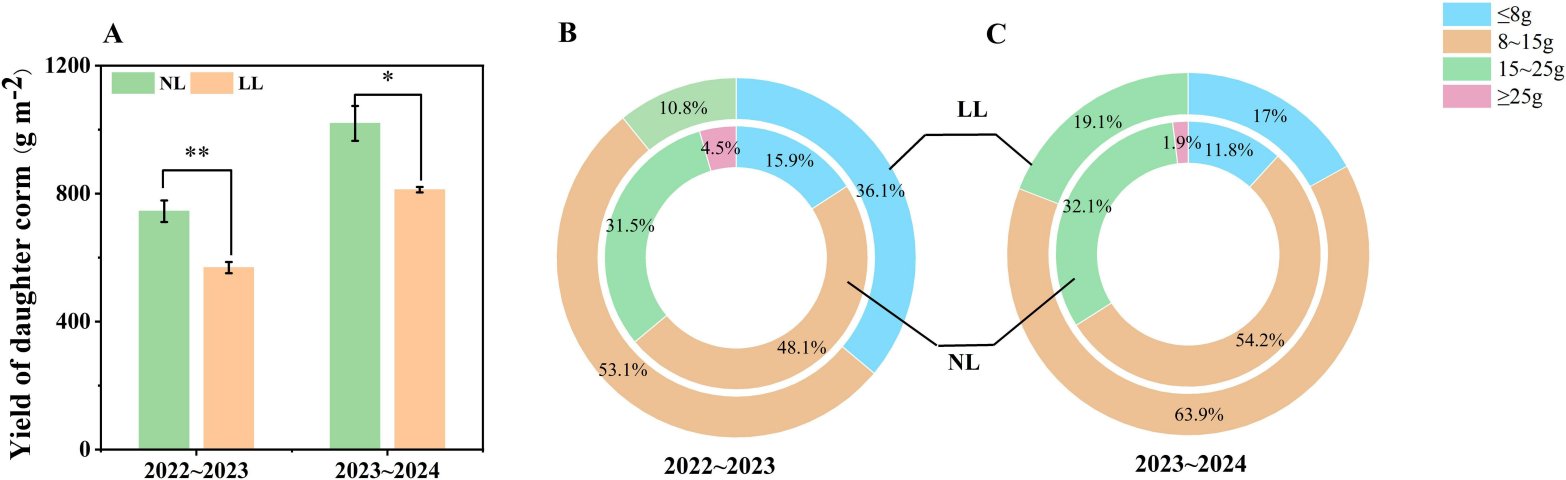
Effect of LL on corm yield **(A)** and yield components **(B, C).** The experiments conducted in 2022~2023 and 2023~2024, and the plants were grown under low light intensity (LL) and natural light intensity (NL). The weights of the four grades grades (≤ 8g, 8~15g, 15~25g, and ≥ 25g) of corms were added together to calculate the total yield. All data were the mean of three replicates. Significant differences between LL and NL treatments were determined using Student’s t-test, *p ≤ 0.05, **p ≤ 0.01.

Daughter corm yield under LL was significantly lower than under NL conditions (**Figure 2A**). Additionally, individual corm weights revealed that LL conditions altered the size distribution of corms. The proportion of small corms (≤8 g) increased under LL, particularly during the 2022~2023 period, while no large corms (≥25 g) were produced in either year (**Figures 2B, C**).

### LL alters leaf morphology and suppresses photosynthetic capacity

**Figure 3:**
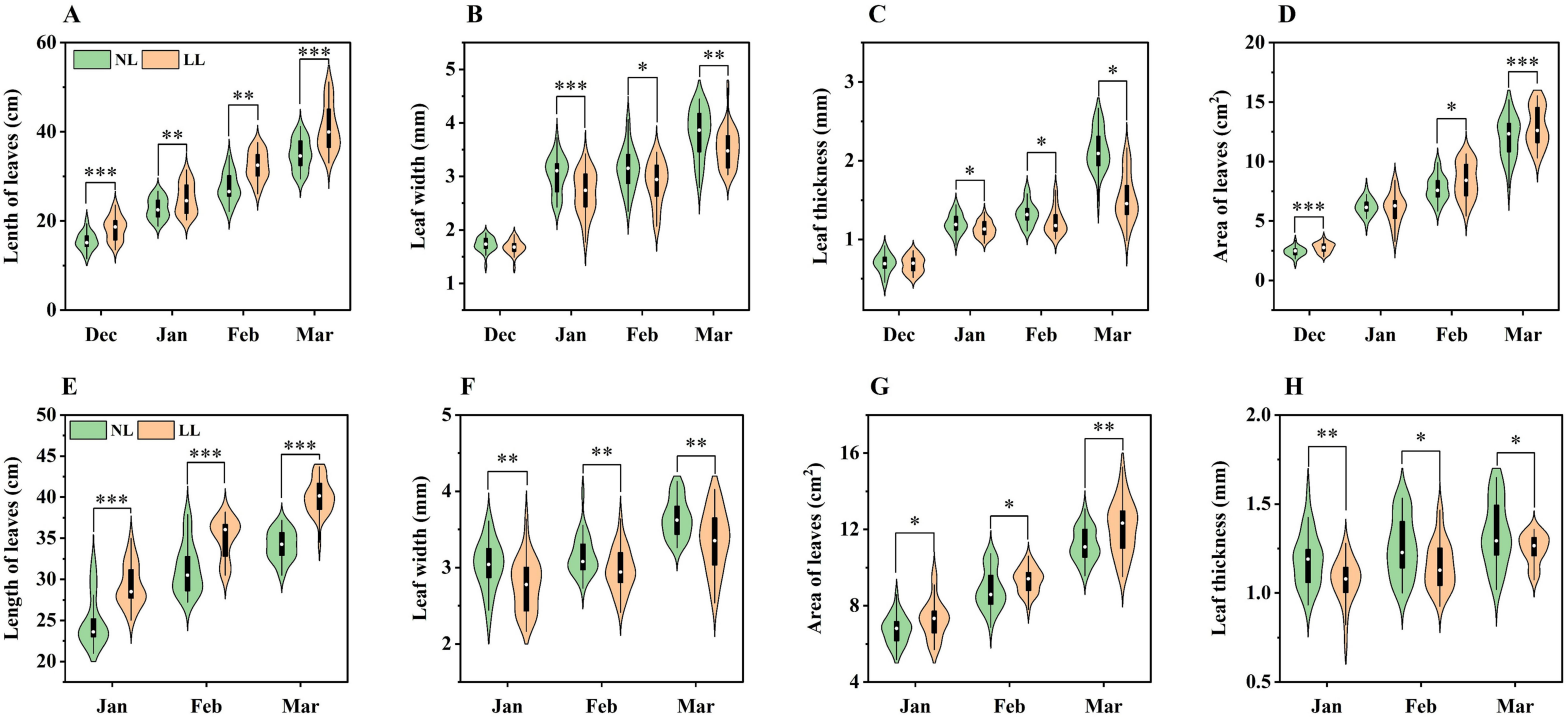
Effect of LL on length **(A, E)**, width **(B, F)**, thickness **(C, G)**, and area **(D, H)** of leaves. The experiments conducted in 2022~2023 **(A–D)** and 2023~2024 **(E–H)**, and the plants were grown under low light intensity (LL) and natural light intensity (NL). Samples were manipulated monthly since December in 2022 and January in 2024. All data were the mean of three replicates. Significant differences between LL and NL treatments were determined using Student’s t-test, *p ≤ 0.05, **p ≤ 0.01, ***p ≤ 0.001.

Under LL conditions, saffron leaves exhibited a marked SAR, with their length and area being significantly greater than those under NL at all sampling times (**Figures 3A, D, E, H**). However, despite their increased size, leaves under LL were narrower and thinner compared to those under NL, with these differences becoming more pronounced during the 2023~2024 growing season (**Figures 3B, C, F, G**).

**Figure 4:**
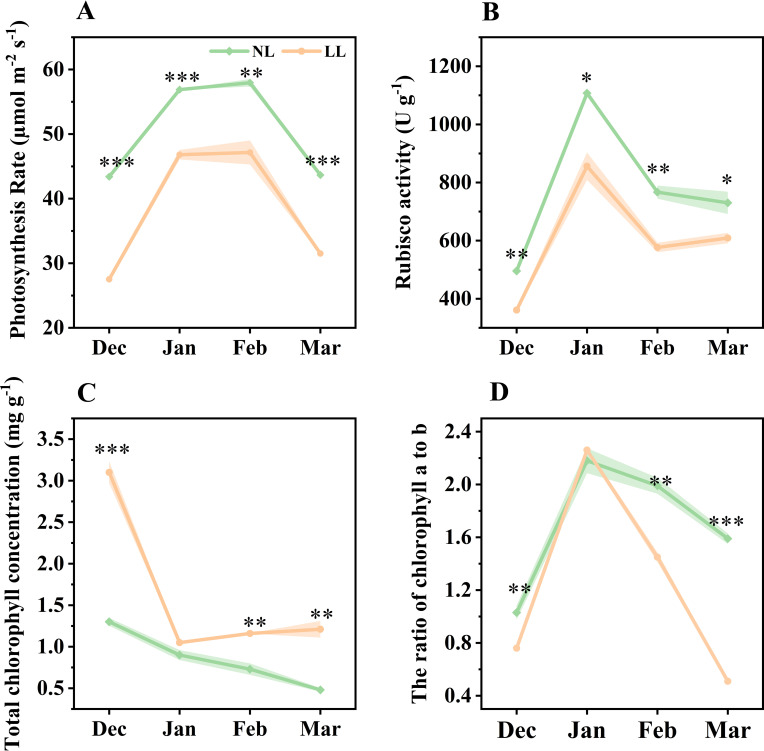
Effect of LL on net photosynthetic rate **(A)**, Rubisco activity **(B)**, total chlorophyll concentration **(C)** and ratio of chlorophyll a to b **(D)**. The experiments conducted in 2022~2023, and the plants were grown under low light intensity (LL) and natural light intensity (NL). Samples were manipulated monthly from December to March of the following year. All data were the mean of three replicates. The shadow besides the line in the figure was the range of standard deviation. Significant differences between LL and NL treatments were determined using Student’s t-test, *p ≤ 0.05, **p ≤ 0.01, ***p ≤ 0.001.

Further analysis focused on the impact of LL on leaf photosynthetic characteristics. The net photosynthetic rate and Rubisco enzyme activity in leaves under LL were significantly lower than those under NL (**Figures 4A, B**). Total chlorophyll concentration increased under LL, yet the ratio of chlorophyll a to b decreased, suggesting an adjustment in the light-harvesting complex (**Figures 4A, B**). Additionally, the light response curve indicated that LL significantly reduced both the maximum net photosynthetic rate and the light saturation point, though it did not affect the dark respiration rate (**Supplementary Figure S2**).

### LL accelerates carbohydrate metabolism in mother corms and inhibits starch synthesis in daughter corm

**Figure 5:**
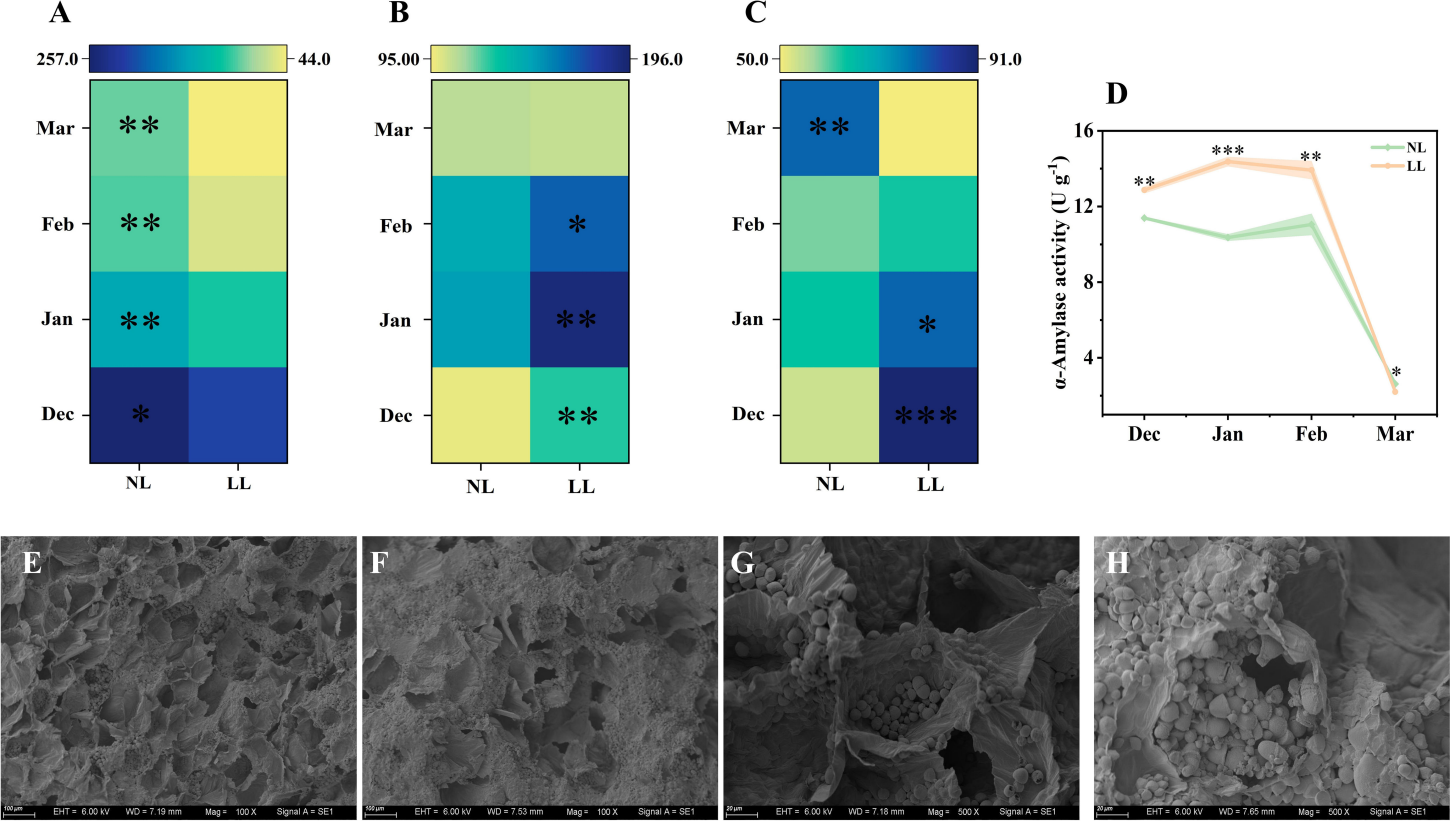
Effect of LL on carbohydrates concentration (mg g^-1^) and activity of α-Amylase **(D)** in the mother corm. The experiments conducted in 2022~2023, and the plants were grown under low light intensity (LL) and natural light intensity (NL). Samples were manipulated monthly from December to March of the following year. All data were the mean of three replicates. The shadow besides the line in the figure was the range of standard deviation. Significant differences between low light LL and NL treatments were determined using Student’s t-test, *p ≤ 0.05, **p ≤ 0.01, ***p ≤ 0.001. **(C–F)**, The morphology of the starch in the mother corm in December was shown in the images taken by scanning electron microscopy. **(A)** starch. **(B)** sucrose. **(C)** glucose. **(E)** starch granules in mother corm in LL (100× magnification). **(F)** starch granules in mother corm in NL (100× magnification). **(G)** starch granules in mother corm in LL (500× magnification). **(H)** starch granules in mother corm in NL (500× magnification). The black holes in the images represent the degradation of starch.

The starch concentration in mother corms under LL was lower than under NL at all sampling times (**Figure 5A**; **Supplementary Figure S4A**). The α-Amylase exhibited significantly higher activity in mother corms under LL conditions (**Figure 5D**). Scanning electron microscopy revealed that starch granules were stored in parenchyma cells, but under LL, the number of empty cell cavities per unit area was significantly higher than under NL (**Figures 5E, F**). Additionally, starch granules in corms under NL were larger and more numerous, whereas granules appeared ruptured under LL (**Figures 5G, H**). These findings suggest that LL accelerates starch degradation in mother corms.

**Figure 6:**
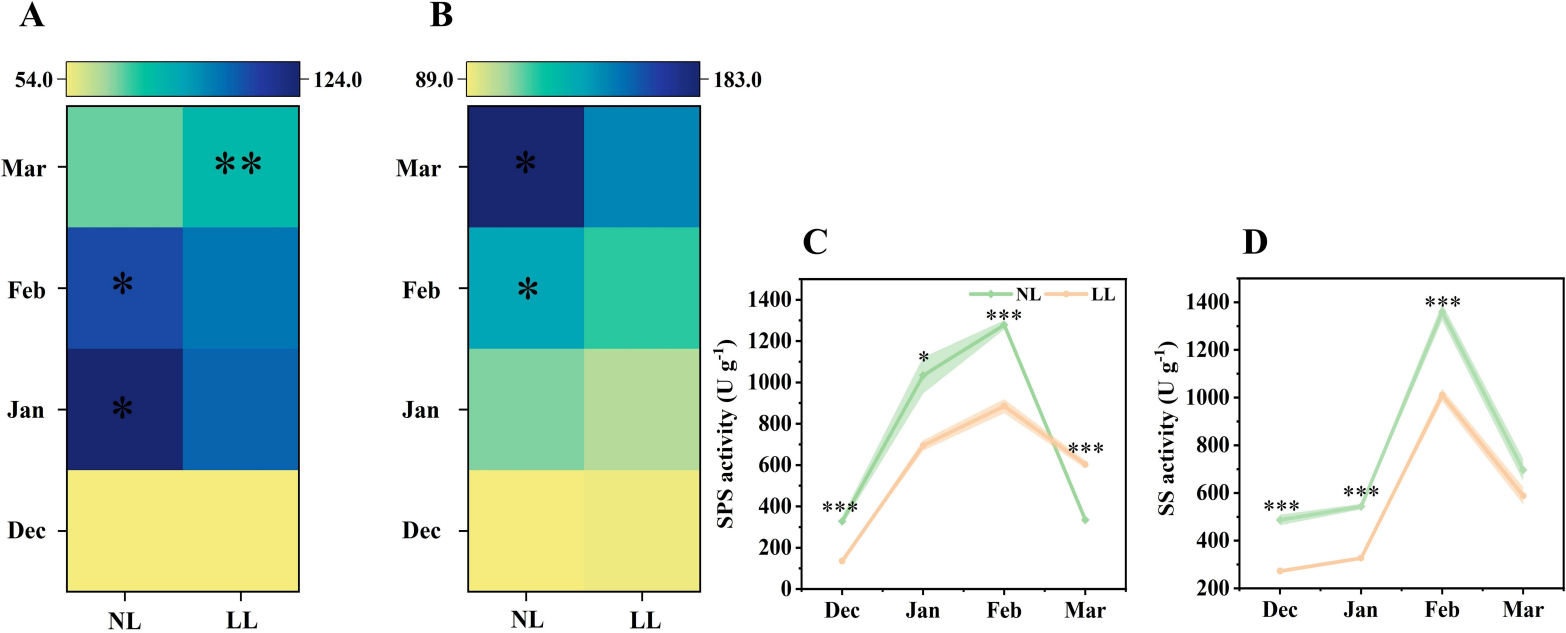
Effect of LL on the concentration of sucrose (mg g^-1^) **(A)** and glucose (mg g^-1^) **(B)** and activity of SPS **(C)** and SS **(D)** in leaves. The experiments conducted in 2022~2023, and the plants were grown under low light intensity (LL) and natural light intensity (NL). Samples were manipulated monthly from December to March of the following year. All data were the mean of three replicates. The shadow besides the line in the figure was the range of standard deviation. SPS, Sucrose phosphate synthase. SS, Sucrose synthase in the synthetic direction. Significant differences between LL and NL treatments were determined using Student’s t-test, *p ≤ 0.05, **p ≤ 0.01, ***p ≤ 0.001.

In March, soluble sugar concentrations in the leaves under NL were higher than those under LL (**Figures 6A, B**; **Supplementary Figure S5**). Glucose concentrations were consistently higher in NL than in LL, and except for March, sucrose concentrations followed a similar trend. The activities of SPS and SS mirrored these changes in sucrose concentration (**Figures 6C, D**).

**Figure 7:**
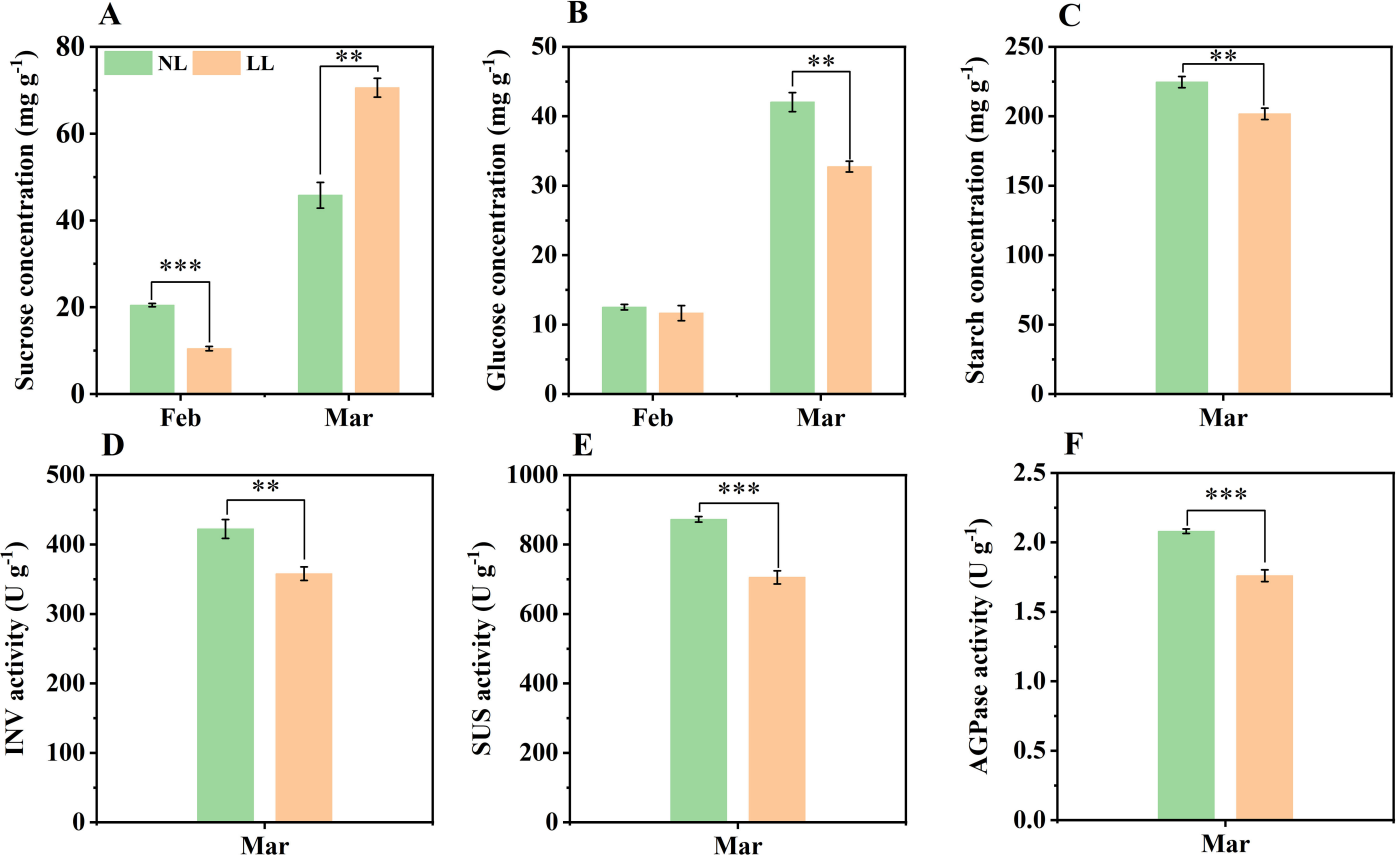
Effect of LL on carbohydrates concentration (mg g^-1^) and activity of INV **(D)**, SUS **(E)** and AGPase **(F)** in daughter corms. The experiments conducted in 2022~2023, and the plants were grown under low light intensity (LL) and natural light intensity (NL). Samples were manipulated monthly from February to March of the following year. All data were the mean of three replicates. **(A)** sucrose. **(B)** glucose. **(C)** starch. INV, Invertase. SUS, Sucrose synthase in the decomposition direction. AGPase, ADP-glucose pyrophosphorylase. Significant differences between LL and NL treatments were determined using Student’s t-test, **p ≤ 0.01, ***p ≤ 0.001.

During daughter corm development, the concentrations of sucrose and glucose gradually increased. By March, the sucrose concentration in daughter corms under LL exceeded that under NL, but both glucose and starch concentrations were lower (**Figures 7A–C**; **Supplementary Figure S6**). The activities of enzymes involved in sucrose hydrolysis, such as INV and SUS, were significantly lower under LL (**Figures 7D, E**). Similarly, AGPase activity of was also inhibited under LL (**Figure 7F**). These results indicate that under low light conditions, sucrose utilization in daughter corms is insufficient, leading to a decrease in glucose, the precursor for starch synthesis, and thereby impairing starch accumulation.

### LL increases IAA and GA_1_ concentrations and decreases ABA concentration

**Figure 8:**
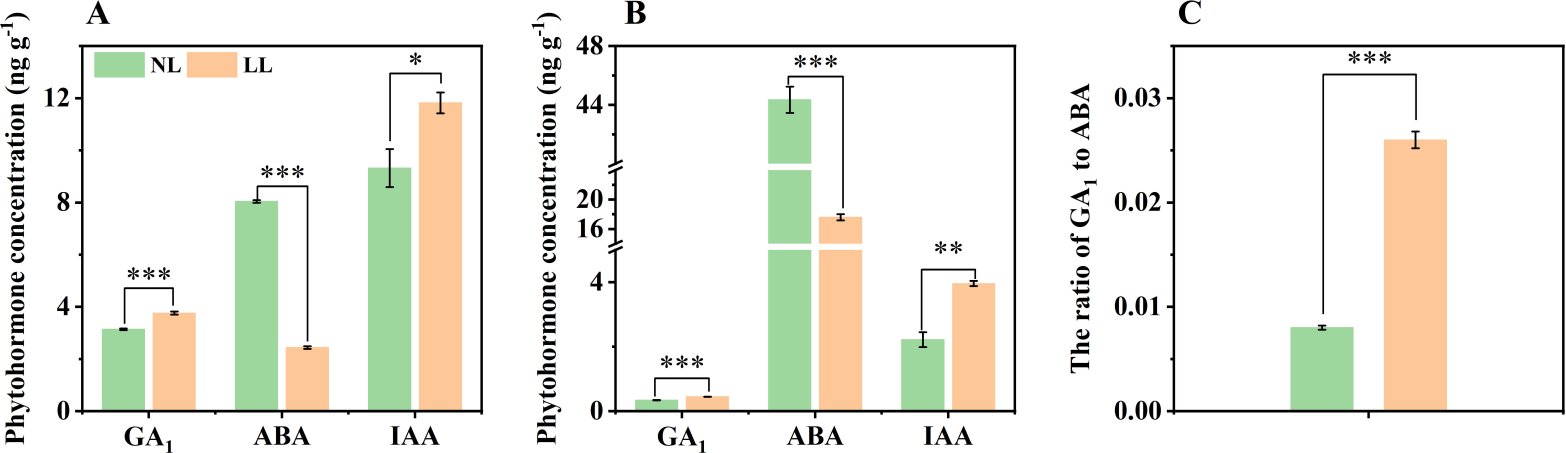
Effect of LL on the concentration of endogenous phytohormones in leaves **(A)** and daughter corms **(B)**, and ratio of GA_1_ to ABA in daughter corms **(C)**. The experiments conducted in 2022~2023, and the plants were grown under low light intensity (LL) and natural light intensity (NL). Samples were manipulated in March of the following year. All data were the mean of three replicates. GA, Gibberellin. ABA, Abscisic acid. IAA, Indole-3-acetic acid (Auxin). Significant differences between LL and NL treatments were determined using Student’s t-test, * *p ≤* 0.05, **p ≤ 0.01, ***p ≤ 0.001.

Endogenous phytohormones play a key role in regulating plant growth and development. In March, the concentrations of GA_1_ and IAA were significantly higher in both leaves and daughter corms under LL compared to NL, while the concentration of ABA was lower under LL (**Figures 8A, B**). Consequently, the GA_1_ to ABA ratio in daughter corms under LL was markedly higher than that under NL, which may be related to the altered carbohydrate metabolism observed under LL (**Figure 8C**). We examined bioactive GAs (GA_1_, GA_3_, GA_4_, and GA_7_) in leaves and daughter corms. Apart from GA_1_, no significant differences were detected in other GAs in leaves and daughter corms, indicating GA_1_ might play a crucial role during the rapid enlargement phase of daughter corms (**Supplementary Table S2**).

### Exogenous GA_3_ application inhibits starch synthase activities in daughter corms

**Figure 9:**
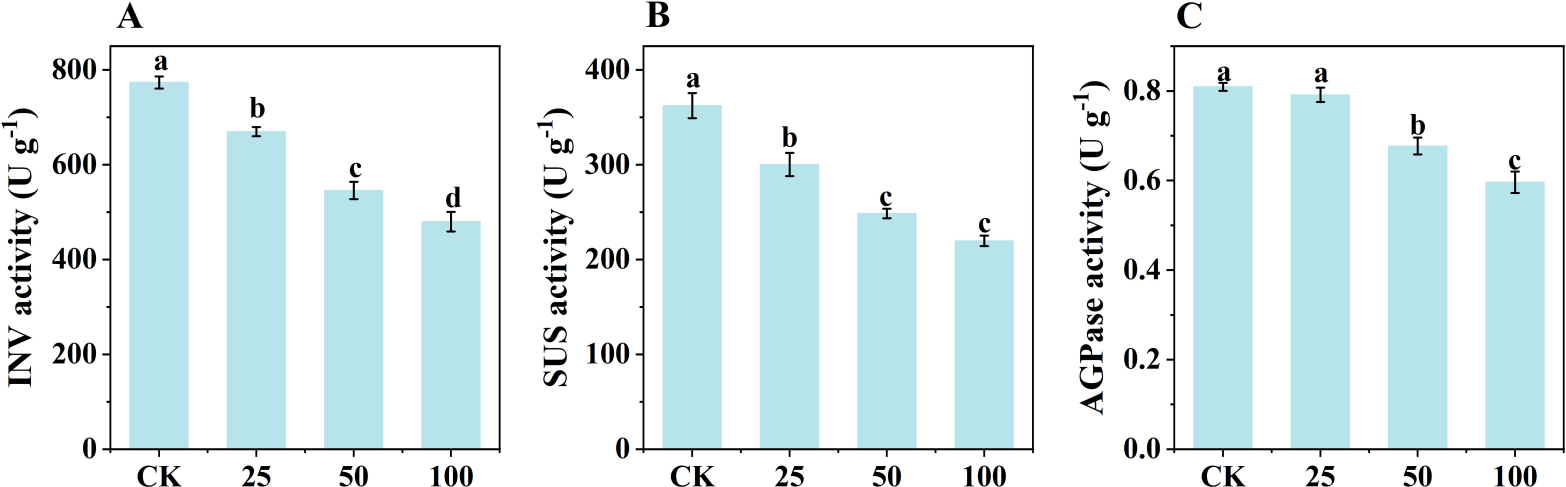
The effect of exogenous GA3 on the activity of INV **(A)**, SUS **(B)** and AGPase **(C)** in daughter corms. The experiment conducted in March, 2024. The plants were grown under natural light intensity (NL). Samples were manipulated one week after GA**_3_** application. All data were the mean of three replicates. CK, 25, 50, 100 represent GA_3_ (μM) application level in control and treatments. INV, Invertase. SUS, Sucrose synthase in the decomposition direction. AGPase, ADP-glucose pyrophosphorylase. Significant differences between LL and NL treatments were determined using One-way ANOVA with significant differences separated by the LSD test at the level of *p ≤* 0.05. Lowercase letters represent significant differences among means were separated according to LSD at the level of *p ≤* 0.05.

To investigate the regulatory effects of phytohormones on starch synthesis, plants grown under NL conditions in March 2024 were treated with GA_3_, and the activities of starch synthesis-related enzymes in daughter corms were measured. GA_3_ application resulted in decreased activities of INV, SUS and AGPase compared to controls. The degree of inhibition was dose-dependent: the higher the GA_3_ concentration, the greater the inhibitory effect on enzyme activities (**Figure 9**).

### RNA-seq reveals differential carbohydrate metabolism pathways in leaves and daughter corms under LL

March is a critical period for corm development, during which significant differences in carbohydrate concentration and enzyme activity were observed between the two light treatments. RNA-seq analysis of leaves and daughter corms in March revealed 6,667 differentially expressed genes (DEGs) in leaves, with 3,318 upregulated and 3,349 downregulated under LL compared to NL. In daughter corms, 2,301 DEGs were identified, with 1,050 upregulated and 1,251 downregulated under LL (**Supplementary Figure S7**).

**Figure 10:**
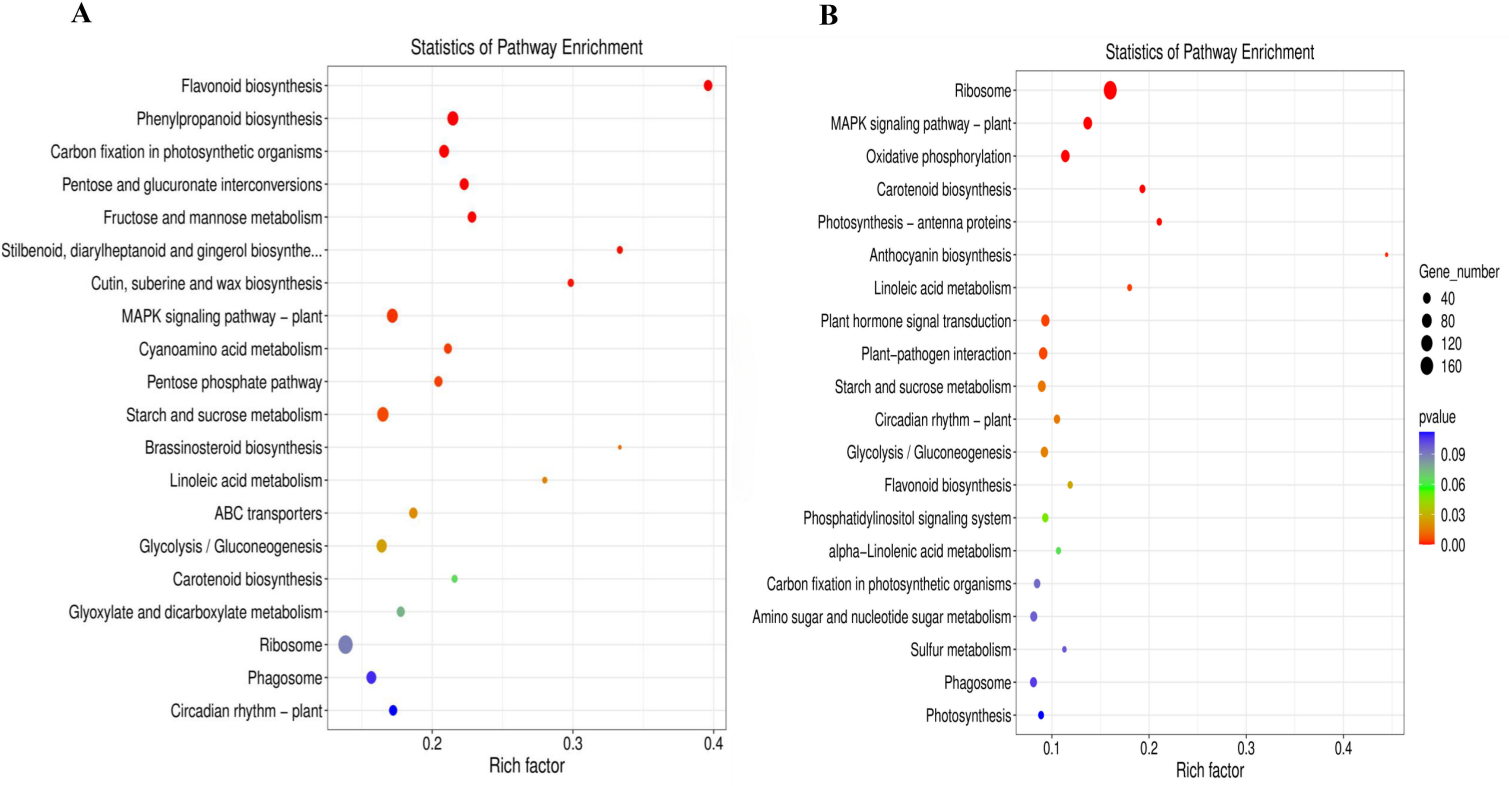
KEGG enrichment analysis in leaves **(A)** and daughter corms **(B)**. The experiments conducted in 2022~2023, and the plants were grown under low light intensity (LL) and natural light intensity (NL). Samples were manipulated in March of the following year. All data were the mean of three replicates. With a threshold of *p ≤* 0.05, the 20 most significantly enriched pathways were selected.

KEGG enrichment analysis revealed 134 and 113 pathways in leaves and daughter corms, respectively, with the top 20 pathways with the lowest *p*-values shown in **Figure 10**. Many DEGs were involved in carbohydrate metabolism, including starch and sucrose metabolism, glycolysis, and other pathways (**Figures 10A, B**). These results suggest that under shading conditions, the expression of carbohydrate metabolic-related genes is more active in leaves than in daughter corms. Relevant DEGs are listed in **Supplementary Tables S3–S5**.

### Carbohydrate metabolism pathway are activated in leaves and inhibited in daughter corms under LL

**Figure 11:**
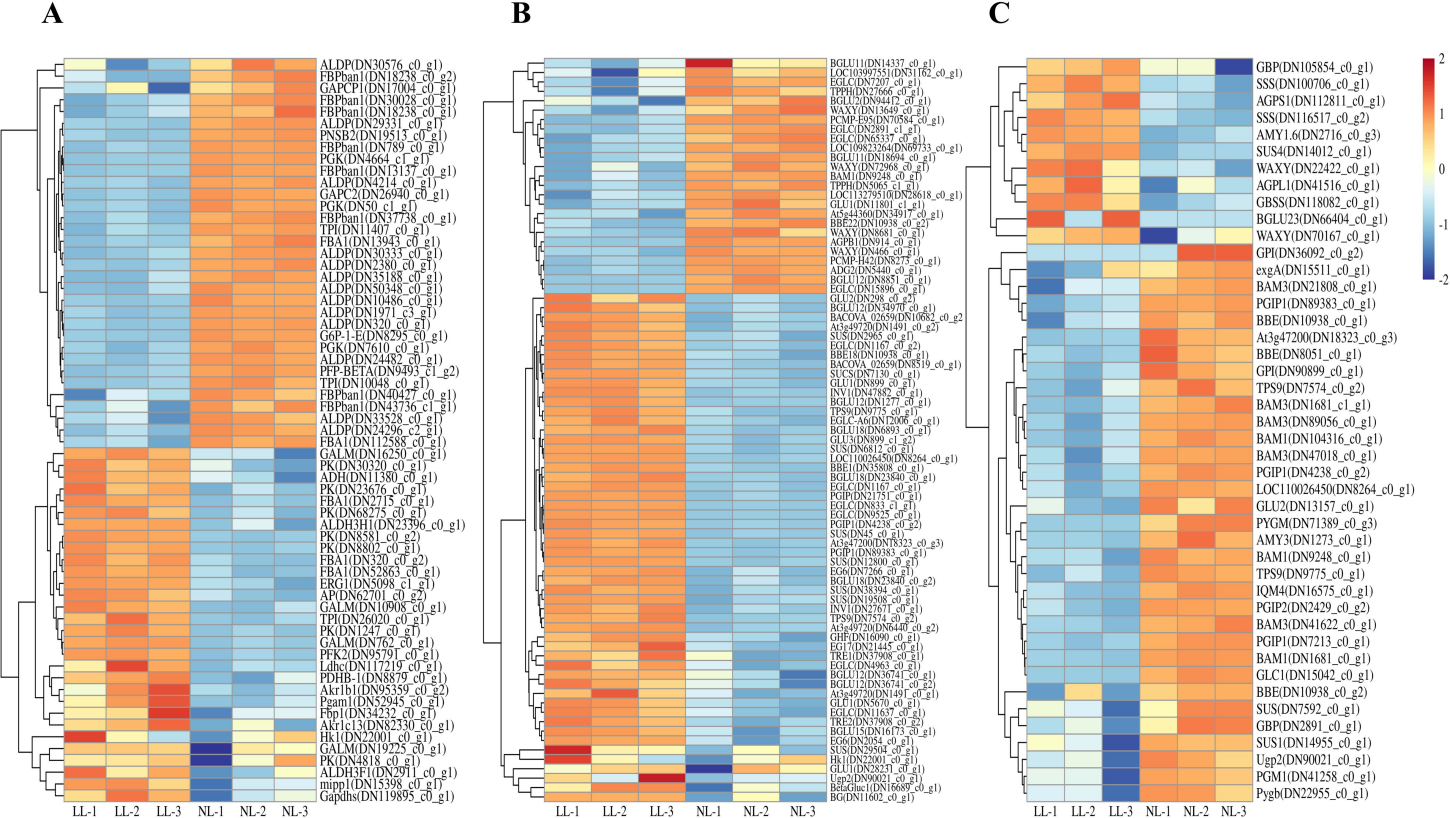
Effect of LL on gene expression of glycolysis **(A)** and starch and sucrose metabolism pathway in leaves **(B)** and sucrose metabolism pathway in daughter corms **(C)**. The experiments conducted in 2022~2023, and the plants were grown under low light intensity (LL) and natural light intensity (NL). Samples were manipulated in March of the following year. All data were the mean of three replicates. The expression levels of the genes have been normalized to TPM values.

Glycolysis, a central pathway for sucrose metabolism and respiration, was significantly upregulated in leaves under LL. A total of 64 DEGs were identified in this pathway, 30 of which were upregulated (**Figure 11A**). Key genes in this pathway, such as *CsHk1* (TRINITY_DN22001_c0_g1), *CsPFK2* (TRINITY_DN95791_c0_g1), and *CsPK* (TRINITY_DN30320_c0_g1), exhibited significant upregulation. Additionally, in the starch and sucrose metabolism pathways, key sucrose hydrolysis enzyme genes, such as *CsINV1* (TRINITY_DN27671_c0_g1) and *CsSUS* (TRINITY_DN29504_c0_g1), also showed varying degrees of upregulation (**Figure 11B**). This indicates that low light activates sugar metabolism pathways in leaves, accelerating sucrose breakdown.

In daughter corms, 44 DEGs related to starch and sucrose metabolism were identified, 33 were downregulated under LL (**Figure 11C**). Unlike in leaves, sucrose hydrolysis enzyme genes, such as *CsSUS* (TRINITY_DN7592_c0_g1), were downregulated in daughter corms. Interestingly, genes involved in starch synthesis, such as *CsAGPS1* (TRINITY_DN41516_c0_g1) and *CsSS1* (TRINITY_DN116517_c0_g2), were upregulated under LL, which was inconsistent with enzyme activity measurements.

### Validation of key gene expression in the carbohydrate metabolism pathway

**Figure 12:**
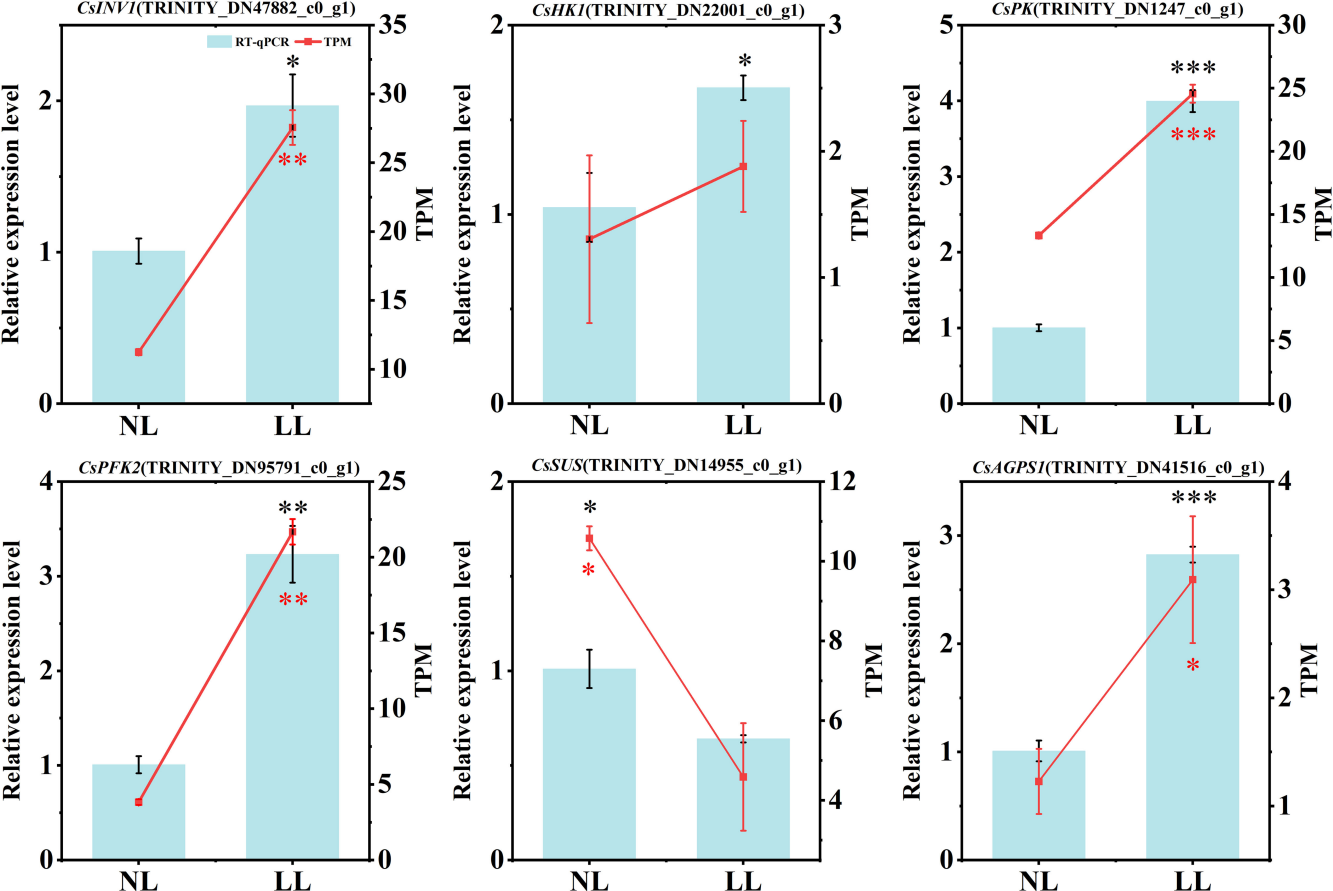
Key gene expression in daughter corms and leaves. The experiments conducted in 2022~2023, and the plants were grown under low light intensity (LL) and natural light intensity (NL). Gene expression was determined using qPCR. Left y-axis represents the relative expression level of genes, right y-axis represents the gene expression level normalized using TPM method. All data were the mean of three replicates. Significant differences in PCR data between LL and NL treatments were determined using Student’s t-test, *p ≤ 0.05, **p ≤ 0.01, ***p ≤ 0.001, black represents the qPCR results, red represents the normalized TPM values of gene expression levels.

To validate the transcriptome data, key DEGs involved in carbohydrate metabolism pathway were selected for qRT-PCR analysis, including *CsINV1* (TRINITY_DN47882_c0_g1), *CsHK1* (TRINITY_DN22001_c0_g1), *CsPK* (TRINITY_DN1247_c0_g1), *CsPFK* (TRINITY_DN95791_c0_g1), *CsSUS* (TRINITY_DN14955_c0_g1) and *CsAGPS1* (TRINITY_DN41516_c0_g1) (**Figure 12**). The results showed that *CsINV1*, *CsHK1*, *CsPK* and *CsPFK* were upregulated in leaves under LL, while the expression of *CsSUS* and *CsAGPS1* were downregulated in daughter corms under LL. These results were consistent with the transcriptome data.

## Discussion

### LL reduces carbohydrate transport from mother corm to daughter corms

The efficient transport of carbohydrates from storage organs to developing organs is a critical factor in determining plant growth and productivity (De Vries et al., 1983; Aguirre et al., 2018). Under LL conditions, our study indicates that carbohydrate allocation in saffron shifts, favoring leaves over developing daughter corms. This shift likely arises from the increased energy demands of expanding leaves in reduced light, prioritizing aboveground growth.

Storage organs such as seeds, roots, and corms serve as primary sources during the transition from heterotrophic to autotrophic growth (Chiariello et al., 1989; Kidson and Westoby, 2000; Franková et al., 2003). This energy allocation supports the establishment of leaf morphology and photosynthetic capacity, facilitating the transition from heterotrophic to autotrophic states. In low light conditions, diminished photosynthetic capacity prolongs reliance on stored carbohydrates (Turgeon, 1989; Krapp and Stitt, 1995; Smith and Stitt, 2007), leading to increased energy allocation to leaves (Poorter and Nagel, 2000; Paul and Foyer, 2001). This is reflected in the higher ratio of leaf to mother corm DW observed under LL (**Figures 1G, I**), aligning with findings in other species where shoot-to-root ratios increase under low light (Poorter and Nagel, 2000). Photosynthetic analysis confirmed reduced leaf photosynthetic capacity under LL, with decreases in PSII operating efficiency, electron transfer rate and photochemical quenching coefficient (**Figure 4A**; **Supplementary Figure S3**). Despite reduced photosynthetic efficiency, no significant differences in leaf DW or soluble sugar concentrations were noted one month after transplanting, indicating that mother corms sufficiently supplied carbohydrates to leaves (**Figures 1B, E**, **4A**, **6A, B**; **Supplementary Figure S5**). Saffron leaf development typically lasts for 3 to 4 months, with energy derived from both photosynthesis and reserves stored in the mother corm (Fallahi and Mahmoodi, 2018; Koocheki and Seyyedi, 2019; Jose-Santhi et al., 2024). When photosynthesis is inhibited, the leaves become increasingly dependent on carbohydrates supplied by the mother corm.

**Figure 13:**
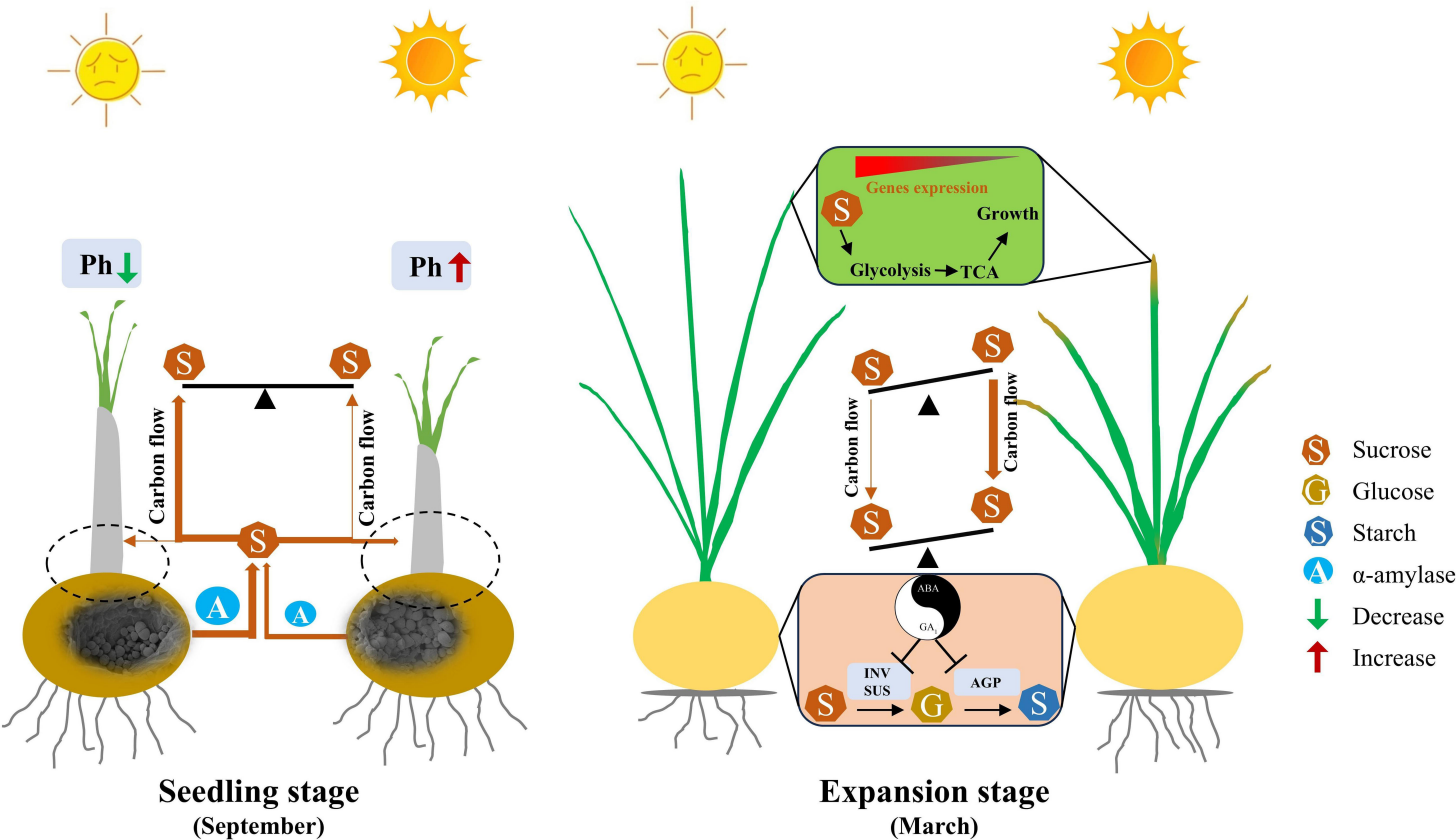
The proposed model for how low light decreases saffron daughter corm yield. At seedling stage, low light reduces the photosynthetic capacity of leaves, accelerating the breakdown of starch into sucrose in mother corms to support leaf growth. This decreases the proportion of carbohydrates allocated to daughter corms, ultimately reducing yield. The dashed line represents the virtual daughter corm that has not yet formed at the seedling stage. During daughter corm enlargement stage, leaves maintain vigorous respiratory metabolism to sustain growth. Concurrently, low light upregulates GA_1_ concentration and downregulates ABA concentration in daughter corms, inhibiting sucrose hydrolysis and starch synthesis, thereby weakening sink intensity. The combined effects of vigorous leaf growth and weakened daughter corm sink strength inhibit sucrose transport from leaves to daughter corms, reducing corm yield. Brown arrows, the amount of carbon flow. T-shaped arrows, the inhibiting effect. Balance scale, the relative sucrose concentration under different light conditions, the side with higher concentration tilts upward, and the side with lower concentration tilts downward. Smile symbol, natural light condition. Sad face symbol, low light condition. GA_1_, Gibberellin 1. ABA, Abscisic acid. Ph, Photosynthesis. INV, Invertase. SUS, Sucrose synthase in the decomposition direction. AGP, ADP-glucose pyrophosphorylase.

Furthermore, under LL, mother corm starch concentration declined markedly, accompanied by increased activity of starch-degrading enzymes such as α-Amylase (**Figures 5A, D**; **Supplementary Figure S4A**). This suggests accelerated starch breakdown in the mother corm to meet the energy needs of expanding leaves. Although the specific proportion of carbohydrates transferred from mother to daughter corms under LL was not measured, the increased carbohydrate allocation to leaves and reduced daughter corm yield indicate diminished transport to daughter corms, contributing to lower yields (**Figure 13**). This outcome is consistent with findings in other in other bulbous crops, where environmental stressors lead to imbalanced energy redistribution and reduced offspring yield (Chawdhry and Sagar, 1973; Verdaguer et al., 2010).

### LL inhibits energy metabolism in daughter corms and carbohydrate transport from leaves

Plants exposed to low light typically display a SAR, allocating more photosynthates to aboveground organs (Givnish, 1988; Niinemets, 2007). However, the specific mechanisms reducing carbohydrate allocation to underground organs under LL remain unclear. According to the pressure-flow model, the transport carbohydrates between source and sink organs depends on their relative strength (Lemoine et al., 2013). During the grain-filling period in crops like rice and wheat, low light weakens the carbohydrate metabolic capacity in grains, reducing sink strength and the total carbohydrate transported from source to sink (Schnyder, 1993; Li et al., 2022; Panda et al., 2023).

In this study, saffron leaves under LL exhibited elongation, thinning and reduced photosynthetic capacity (**Figures 3**, **4**, **6**), consistent with SAR observed in other species (Gregoriou et al., 2007; Yao et al., 2017). However, sucrose concentration in daughter corms was higher under LL than under NL during daughter corm enlargement (**Figure 7A**; **Supplementary Figure S6A**). Sucrose, the primary form of carbohydrate transport, is hydrolyzed to glucose in the corm and catalyzed by enzymes to synthesize starch (Quick and Schaffer, 2017; Ma et al., 2021). Despite higher sucrose levels, both starch concentration and corm yield decreased under LL (**Figures 2**, **7C**; **Supplementary Figures S6B, C**). Under LL, sucrose accumulated in daughter corms, while glucose concentration was lower than under NL (**Figures 7A, B**). The activities of key enzymes involved in sucrose hydrolysis and starch synthesis—SUS and INV, were also inhibited under LL (**Figures 7D, E**). Transcriptional analysis revealed a significant downregulation of genes associated with sucrose and starch metabolism, including a 56.63% reduction in SUS gene expression (**Figures 11C**, **12**). These results indicate that LL inhibits carbohydrate metabolism in saffron daughter corms. There is an observed inconsistency between the enzymatic activities and encoding gene transcript levels of AGPase in daughter corms under LL (**Figures 7F**, **12**). AGPase activity is regulated by a variety of mechanisms, including transcriptional regulation (Mangelsen et al., 2010), allosteric interactions (Geigenberger, 2003), and protein phosphorylation (Yu et al., 2023a). In starch-storing tissues, the enzyme activity of AGPase has been found to correlate positively with the phosphorylation of its subunits (Ferrero et al., 2020; Yu et al., 2023b). Further studies are required to understand the discrepancy between AGPase enzymatic activity and the expression of its encoding genes in saffron corms.

The ratio of carbohydrate concentration between organs can serve as an indicator of the distribution of carbohydrate among organs (Friend et al., 1994; He et al., 2020; Zhou et al., 2021). For example, under low light conditions, the ratio of leaves to roots sucrose in soybean is significantly higher than under natural light conditions, indicating that a higher proportion of sucrose is retained in the leaves rather than transported to the roots (Feng et al., 2018; Zhou et al., 2019). Our data are consistent with this observation, the ratio of leaf to corm sucrose under LL was higher than under NL. These results demonstrated that LL reduces the ability of saffron daughter corms to metabolize sucrose, inhibiting carbohydrate transport from leaves and resulting in insufficient substrate for starch synthesis, thus reducing corm yield.

During saffron corm rapid enlargement period, more carbohydrates were kept in leaves under LL than NL (**Figures 1G–J**). Unlike many annual plants that prioritize reproductive organs under low light (McMaster et al., 1987; Li et al., 2010; Dong et al., 2014; Keller et al., 2021; Liang et al., 2023), and differing from saffron plants grown in Iran, where leaf biomass gradually decreases as daughter corms expand and the timing of leaf development and corm rapid enlargement does not overlap (Renau-Morata et al., 2012; Behdani and Fallahi, 2015; Fallahi and Mahmoodi, 2018). Here, Saffron leaves did not undergo premature senescence and instead maintained active growth under LL (**Figures 1B, E**, **3**). The higher auxin concentration observed in LL-treated leaves, alongside upregulation of genes related to respiratory metabolism, suggests that leaves continued acting as a significant carbohydrate sinks (**Figure 8A**; **Supplementary Figure S8**) (Gören-Saglam et al., 2020; Tivendale and Millar, 2022). Possible explanations for the distinct response of saffron leaves to LL, compared to annual plants during their reproductive phase, are: 1) Although the corm is a reproductive organ, it is not a seed-producing organ, which may alter the plant’s resource allocation strategy. 2) The strength of the sink signal regulating leaf growth, particularly in terms of energy demand signals (Marcelis et al., 2004; McCormick et al., 2009), may be insufficient, leading to a prolonged leaf development phase (**Figure 13**). Before leaf morphogenesis is complete, the aboveground part is the plant’s growth center, resulting in more carbohydrates being allocated to the growth center (Smith and Stitt, 2007). The unique growth and development pattern of saffron, where leaf morphogenesis overlaps with the development of daughter corms, leads to reduced carbohydrate allocation to daughter corms under LL conditions. This prolonged developmental overlap between leaves and daughter corms likely delayed the transition of leaves from sink to source, prolonging competition for carbohydrates and contributing to reduced carbohydrate allocation to daughter corms and lower yields (**Figure 13**).

### LL modulates carbohydrate metabolism enzymes via GAs and ABA

Our study revealed that LL conditions significantly alter the balance between GAs and ABA in saffron daughter corms, inhibiting the activity of key carbohydrate metabolism enzymes. Plant phytohormones like GAs and ABA play crucial roles in carbohydrate metabolism during underground organ expansion (Chen et al., 2022b). ABA positively affects tuber formation by counteracting the effects of GAs. For instance, during potato (*Solanum tuberosum* L.) tuber enlargement, an increase in ABA concentration and a decrease in GAs concentration are observed, and the use of the GAs inhibitor tetcyclacis can prevent tuber death in ABA-deficient droopy potato mutants (Vreugdenhil et al., 1994; Xu et al., 1998). In gladiolus, GA_3_ and ABA antagonistically regulate starch synthesis, with GA_3_ inhibiting corm development by reducing the expression of *GhSUS* (Li et al., 2021).

In March, the concentration of GA_1_ in daughter corms was higher under LL (**Figure 8**), consistent with results from sweet potato research and our previous results in saffron, where root GA_3_ concentration was significantly higher after 40 days of 40% shade compared to natural light conditions (Wang et al., 2014; Zhou et al., 2024). However, LL reduced the ABA biosynthesis, resulting in increases in the ratio of GA_1_ to ABA (**Figure 8**). The activities of SUS, INV, and AGPase in corms were lower under LL (**Figures 7D–F**). Based on these results, we hypothesis that LL upregulates the endogenous ratio of GA_1_ to ABA in saffron daughter corms, thereby inhibiting the activity of enzymes involved in carbohydrate metabolism (**Figure 13**).

Exogenous GA_3_ application inhibited SUS, INV, and AGPase enzyme activity (**Figure 9**), consistent with results reported in potato and gladiolus (Wang et al., 2014; Li et al., 2021). Further, the gene transcription analysis also supported the phytohormone quantification data (**Figure 11**). Specifically, the rate-limiting enzymes for ABA synthesis, *CsZEPs* and *CsNCED*, were downregulated by 1.56-1.63 fold and 1.11 fold under LL, respectively (**Supplementary Figure S9**; **Supplementary Table S6**), this is consistent with the results reported in tomato (*Lycopersicon esculentum* cv. Ailsa Craig) and barley (*Hordeum vulgare* L.) that low light may decrease ABA concentration by inhibiting carotenoid synthesis (Thompson et al., 2000; Rikiishi et al., 2015). These results confirmed that during the expansion stage of saffron daughter corms, LL inhibits ABA synthesis, increases the concentration ratio of GA_1_ to ABA, and decreases the activity of carbohydrate metabolism enzymes, ultimately adversely affecting starch synthesis and corm formation (**Figure 13**). Although our study elucidates the role of hormonal regulation in response to low light, further research is needed to fully understand the molecular mechanisms involved in light-mediated regulation of starch synthesis in saffron corms.

## Conclusion

This study provides insights into the effects of LL conditions on carbohydrate allocation and metabolism in saffron corms. Under LL, saffron displayed altered carbohydrate distribution, prioritizing leaf growth over the development of daughter corms. This shift was associated with decreased photosynthetic efficiency and increased reliance on stored carbohydrates from mother corms, ultimately reducing the yield of daughter corms. Furthermore, LL conditions inhibited key enzymes involved in carbohydrate metabolism in daughter corms, such as SUS and INV, and disrupted the hormonal balance of GAs and ABA, which are crucial for regulating starch synthesis. This metabolic bottleneck in daughter corms resulted in reduced carbohydrate transport from the leaves, exacerbating the decline in corm biomass and yield. The diminished sink strength in daughter corms under LL conditions illustrates how limited carbohydrate metabolism can disrupt the source-sink relationship, hampering the allocation of resources necessary for proper corm development. These findings extend our understanding of how light availability influences carbohydrate allocation strategies in geophytic plants and emphasize the importance of metabolic activity in regulating source-sink dynamics. Understanding these responses is vital not only for optimizing agricultural practices but also for predicting plant performance under fluctuating environmental conditions.

## Data Availability

The datasets presented in this study can be found in online repositories. The names of the repository/repositories and accession number(s) can be found in the article/**Supplementary Material**.

## References

[B1] Aguirre M. Kiegle E. Leo G. Ezquer I. (2018). Carbohydrate reserves and seed development: an overview. Plant Reprod. 31, 263–290. doi: 10.1007/s00497-018-0336-3 29728792

[B2] Ahrazem O. Rubio-Moraga A. Nebauer S. G. Molina R. V. Gomez-Gomez L. (2015). Saffron: its phytochemistry, developmental processes, and biotechnological prospects. J. Agric. Food Chem. 63, 8751–8764. doi: 10.1021/acs.jafc.5b03194 26414550

[B3] Arnon D. I. (1949). Copper enzymes in isolated chloroplasts. Polyphenol. Beta Vulgaris. Plant Physiol. 24, 1–15. doi: 10.1104/pp.24.1.1 PMC437905 16654194

[B4] Behdani M. Fallahi H. (2015). Saffron: Technical knowledge and research-based approaches (Iran, University of Birjand Publication).

[B5] Buchfink B. Xie C. Huson D. H. (2015). Fast and sensitive protein alignment using DIAMOND. Nat. Methods 12, 59–60. doi: 10.1038/nmeth.3176 25402007

[B6] Cardone L. Candido V. Castronuovo D. Perniola M. Cicco N. (2021). Comparing annual and biennial crop cycle on the growth, yield and quality of saffron using three corm dimensions. Sci. Horticult. 288. doi: 10.1016/j.scienta.2021.110393

[B7] Chawdhry M. Sagar G. (1973). An autoradiographic study of the distribution of 14c-labelled assimilates, derived from 14CO2 fed to the aerial parts, was made at ten different developmental stages of *Oxalis latifolia* H.B.K. And *Oxalis Pes-caprae* L. Weed Res. 13, 430–437. doi: 10.1111/j.1365-3180.1973.tb01297.x

[B8] Chen C. Baethgen W. E. Robertson A. (2013). Contributions of individual variation in temperature, solar radiation and precipitation to crop yield in the North China Plain 1961–2003. Climatic Change. 116, 767–788. doi: 10.1007/s10584-012-0509-2

[B9] Chen Z. F. Wang T. H. Feng C. Y. Guo H. F. Guan X. X. Zhang T. L.. (2022a). Multigene manipulation of photosynthetic carbon metabolism enhances the photosynthetic capacity and biomass yield of cucumber under low-CO_2_ environment. Front. Plant Sci. 13. doi: 10.3389/fpls.2022.1005261 PMC9623318 36330244

[B10] Chen P. L. Yang R. X. Bartels D. Dong T. Y. Duan H. Y. (2022b). Roles of abscisic acid and gibberellins in stem/root tuber development. Int. J. Mol. Sci. 23. doi: 10.3390/ijms23094955 PMC9102914 35563355

[B11] Chiariello N. R. Mooney H. A. Williams K. (1989). “Growth, carbon allocation and cost of plant tissues,” in Plant Physiological Ecology: field methods and instrumentation (Berlin: Springer), 327–365. doi: 10.1007/978-94-009-2221-1_15

[B12] De Vries F. P. Van Laar H. Chardon M. C. M. (1983). “Bioenergetics of growth of seeds, fruits and storage organs,” in Potential Productivity of Field Crops Under Different Environments, (Banos: International Rice Research Institute). 37–60.

[B13] Dong C. Fu Y. M. Liu G. H. Liu H. (2014). Low light intensity effects on the growth, photosynthetic characteristics, antioxidant capacity, yield and quality of wheat (*Triticum aestivum* L.) at different growth stages in BLSS. Adv. Space Res. 53, 1557–1566. doi: 10.1016/j.asr.2014.02.004

[B14] Dong W. Q. He F. L. Jiang H. P. Liu L. L. Qiu Z. Y. (2021). Comparative transcriptome sequencing of taro corm development with a focus on the starch and sucrose metabolism pathway. Front. Genet. 12. doi: 10.3389/fgene.2021.771081 PMC8630585 34858484

[B15] Fallahi H. R. Mahmoodi S. (2018). Impact of water availability and fertilization management on saffron (*Crocus sativus* L.) biomass allocation. J. Horticult. Postharvest Res. 1, 131–146. doi: 10.22077/JHPR.2018.1487.1017

[B16] Fang S. Yang C. Ali M. M. Lin M. Tian S. Zhang L.. (2022). Transcriptome analysis reveals the molecular regularity mechanism underlying stem bulblet formation in oriental Lily ‘Siberia’; Functional Characterization of the LoLOB18 Gene. Int. J. Mol. Sci. 23. doi: 10.3390/ijms232315246 PMC9738039 36499579

[B17] Feng L. Raza M. A. Li Z. Chen Y. Khalid M. H. B. Du J.. (2018). The influence of light intensity and leaf movement on photosynthesis characteristics and carbon balance of soybean. Front. Plant Sci. 9. doi: 10.3389/fpls.2018.01952 PMC6338029 30687355

[B18] Ferrero D. M. L. Piattoni C. V. Diez M. D. A. Rojas B. E. Hartman M. D. Ballicora M. A.. (2020). Phosphorylation of ADP-Glucose pyrophosphorylase during wheat seeds development. Front. Plant Sci. 11. doi: 10.3389/fpls.2020.01058 PMC7366821 32754189

[B19] Finley J. W. Gao S. (2017). A perspective on *Crocus sativus* L. (Saffron) constituent crocin: A potent water-soluble antioxidant and potential therapy for Alzheimer’s disease. J. Agric. Food Chem. 65, 1005–1020. doi: 10.1021/acs.jafc.6b04398 28098452

[B20] Franklin K. A. (2008). Shade avoidance. New Phytol. 179, 930–944. doi: 10.1111/j.1469-8137.2008.02507.x 18537892

[B21] Franková L. Komjáthyová H. Bóka K. Gašparíková O. Pšenák M. (2003). Biochemical and physiological aspects of developmental cycle of *Colchicum autumnale* L. Biol. Plant. 47, 509–516. doi: 10.1023/B:BIOP.0000041054.71761.0b

[B22] Friend A. L. Coleman M. D. Isebrands J. (1994). Carbon allocation to root and shoot systems of woody plants. Biol. Adventitious Root Form. 62, 245–273. doi: 10.1007/978-1-4757-9492-2_18

[B23] Gao P. Tian Z. W. Lu Y. Q. Lu M. Zhang H. H. Wu H. R.. (2022). A decision-making model for light environment control of tomato seedlings aiming at the knee point of light-response curves. Comput. Electron. Agric. 198. doi: 10.1016/j.compag.2022.107103

[B24] Ge L. H. Huang Y. L. Li X. Wang N. X. X. Liu J. Q. Liu M. T.. (2024). Temperature-driven divergence in molecular distribution and microbial invasion and the associated texture softening during dual-phase fermentation of Paocai. Food Chem. 457. doi: 10.1016/j.foodchem.2024.140171 38908247

[B25] Geigenberger P. (2003). Regulation of sucrose to starch conversion in growing potato tubers. J. Exp. Bot. 54, 457–465. doi: 10.1093/jxb/erg074 12508056

[B26] Givnish T. J. (1988). Adaptation to sun and shade: a whole-plant perspective. Funct. Plant Biol. 15, 63–92. doi: 10.1071/pp9880063

[B27] Gören-Saglam N. Harrison E. Breeze E. Öz G. Buchanan-Wollaston V. (2020). Analysis of the impact of indole-3-acetic acid (IAA) on gene expression during leaf senescence in. Physiol. Mol. Biol. Plants. 26, 733–745. doi: 10.1007/s12298-019-00752-7 32255936 PMC7113346

[B28] Grabherr M. G. Haas B. J. Yassour M. Levin J. Z. Thompson D. A. Amit I.. (2011). Full-length transcriptome assembly from RNA-Seq data without a reference genome. Nat. Biotechnol. 29, 644–652. doi: 10.1038/nbt.1883 21572440 PMC3571712

[B29] Gregoriou K. Pontikis K. Vemmos S. (2007). Effects of reduced irradiance on leaf morphology, photosynthetic capacity, and fruit yield in olive (*Olea europaea* L.). Photosynthetica 45, 172–181. doi: 10.1007/s11099-007-0029-x

[B30] Gresta F. Lombardo G. Siracusa L. Ruberto G. (2009). Saffron, an alternative crop for sustainable agricultural systems: a review. Sustain. Agric. Rev. 28, 95–112. doi: 10.1051/agro:2007030

[B31] Hawker J. Marschner H. Krauss A. (1979). Starch synthesis in developing potato tubers. Physiol. Plant. 46, 25–30. doi: 10.1111/j.1399-3054.1979.tb03180.x

[B32] He W. Q. Liu H. Y. Qi Y. Liu F. Zhu X. R. (2020). Patterns in nonstructural carbohydrate contents at the tree organ level in response to drought duration. Global Change Biol. 26, 3627–3638. doi: 10.1111/gcb.15078 32162388

[B33] Jose-Santhi J. Sheikh F. R. Kalia D. Sood R. Kumar R. Acharya V.. (2024). Transcriptional dynamics in source-sink tissues identifies molecular factors regulating the corm development process in saffron (*Crocus sativus* L.). Physiol. Plant. 176. doi: 10.1111/ppl.14285 38606764

[B34] Ke W. J. Li Y. R. Zhong F. R. Pen M. Y. Dong J. J. Xu B. J.. (2023). Relatively high light inhibits reserves degradation in the Coptis chinensis rhizome during the leaf expansion by changing the source-sink relationship. Front. Plant Sci. 14. doi: 10.3389/fpls.2023.1225895 PMC10502731 37719221

[B35] Keller I. Rodrigues C. M. Neuhaus H. E. Pommerrenig B. (2021). Improved resource allocation and stabilization of yield under abiotic stress. J. Plant Physiol. 257. doi: 10.1016/j.jplph.2020.153336 33360492

[B36] Khodakarami J. Ghobadi P. (2016). Urban pollution and solar radiation impacts. Renewable Sustain. Energy Rev. 57, 965–976. doi: 10.1016/j.rser.2015.12.166

[B37] Kidson R. Westoby M. (2000). Seed mass and seedling dimensions in relation to seedling establishment. Oecologia 125, 11–17. doi: 10.1007/PL00008882 28308213

[B38] Kikuzawa K. (1995). The basis for variation in leaf longevity of plants. Vegetatio 121, 89–100. doi: 10.1007/BF00044675

[B39] Kitajima K. J. O. (1994). Relative importance of photosynthetic traits and allocation patterns as correlates of seedling shade tolerance of 13 tropical trees. Oecologia 98, 419–428. doi: 10.1007/bf00324232 28313920

[B40] Koch K. (1996). Carbohydrate-modulated gene expression in plants. Annu. Rev. Plant Biol. 47, 509–540. doi: 10.1146/annurev.arplant.47.1.509 15012299

[B41] Koocheki A. Seyyedi S. M. (2019). Mother corm origin and planting depth affect physiological responses in saffron (*Crocus sativus* L.) under controlled freezing conditions. Ind. Crops Prod. 138. doi: 10.1016/j.indcrop.2019.111468

[B42] Krapp A. Stitt M. (1995). An evaluation of direct and indirect mechanisms for the “sink-regulation” of photosynthesis in spinach: changes in gas exchange, carbohydrates, metabolites, enzyme activities and steady-state transcript levels after cold-girdling source leaves. Planta 195, 313–323. doi: 10.1007/bf00202587

[B43] Kumar A. Devi M. Kumar R. Kumar S. (2022). Introduction of high-value *Crocus sativus* (saffron) cultivation in non-traditional regions of India through ecological modelling. Sci. Rep. 12. doi: 10.1038/s41598-022-15907-y PMC9279281 35831447

[B44] Lemoine R. La Camera S. Atanassova R. Déedaldéechamp F. Allario T. Pourtau N.. (2013). Source-to-sink transport of sugar and regulation by environmental factors. Front. Plant Sci. 4. doi: 10.3389/fpls.2013.00272 PMC3721551 23898339

[B45] Li Q. P. Deng F. Zeng Y. L. Li B. He C. Y. Zhu Y. Y.. (2022). Low light stress increases chalkiness by disturbing starch synthesis and grain filling of rice. Int. J. Mol. Sci. 23. doi: 10.3390/ijms23169153 PMC9408977 36012414

[B46] Li H. Jiang D. Wollenweber B. Dai T. Cao W. (2010). Effects of shading on morphology, physiology and grain yield of winter wheat. Eur. J. Agro. 33, 267–275. doi: 10.1016/j.eja.2010.07.002

[B47] Li J. R. Seng S. S. Li D. L. Zhang F. Q. Liu Y. X. Yao T.. (2021). Antagonism between abscisic acid and gibberellin regulates starch synthesis and corm development in. Horticult. Res. 8,2126–2137. doi: 10.1038/s41438-021-00589-w PMC8245626 34193854

[B48] Liang X. G. Gao Z. Fu X. X. Chen X. M. Shen S. Zhou S. L. (2023). Coordination of carbon assimilation, allocation, and utilization for systemic improvement of cereal yield. Front. Plant Sci. 14. doi: 10.3389/fpls.2023.1206829 PMC10508850 37731984

[B49] Liang F. Xia X. (2005). Long-term trends in solar radiation and the associated climatic factors over China for 1961-2000. Annales Geophys. 23, 2425–2432. doi: 10.5194/angeo-23-2425-2005

[B50] Liu X. W. Sun B. B. Xu C. Y. Zhang T. X. Zhang Y. Q. Zhu L. Y. (2023). Intrinsic mechanisms for the inhibition effect of graphene oxide on the catalysis activity of alpha amylase. J. Hazard. Mater. 453. doi: 10.1016/j.jhazmat.2023.131389 37043854

[B51] Lundmark M. Hurry V. Lapointe L. (2009). Low temperature maximizes growth of *Crocus vernus* (L.) Hill via changes in carbon partitioning and corm development. J. Exp. Bot. 60, 2203–2213. doi: 10.1093/jxb/erp103 19403850 PMC2682509

[B52] Ma L. Ding S. Fu X. Yan Z. Tang D. (2021). Enzymatic and transcriptomic analysis reveals the essential role of carbohydrate metabolism in freesia (*Freesia hybrida*) corm formation. PeerJ 9, e11078. doi: 10.7717/peerj.11078 33777537 PMC7983857

[B53] Mangelsen E. Wanke D. Kilian J. Sundberg E. Harter K. Jansson C. (2010). Significance of light, sugar, and amino acid supply for diurnal gene regulation in developing barley caryopses. Plant Physiol. 153, 14–33. doi: 10.1104/pp.110.154856 20304969 PMC2862414

[B54] Marcelis L. F. Heuvelink E. Hofman-Eijer L. R. Bakker J. D. Xue L. B. (2004). Flower and fruit abortion in sweet pepper in relation to source and sink strength. J. Exp. Bot. 55, 2261–2268. doi: 10.1093/jxb/erh245 15333643

[B55] McCormick A. J. Watt D. A. Cramer M. D. (2009). Supply and demand: sink regulation of sugar accumulation in sugarcane. J. Exp. Bot. 60, 357–364. doi: 10.1093/jxb/ern310 19050062

[B56] McMaster G. S. Morgan J. A. Willis W. O. (1987). Effects of Shading on Winter Wheat Yield, Spike characteristics, and carbohydrate allocation 1. Crop Sci. 27, 967–973. doi: 10.2135/cropsci1987.0011183X002700050030x

[B57] Mzabri I. Addi M. Berrichi A. J. C. (2019). Traditional and modern uses of saffron (Crocus sativus). Cosmetics. 6, 63. doi: 10.3390/cosmetics6040063

[B58] Niinemets U. (2007). Photosynthesis and resource distribution through plant canopies. Plant Cell Environ. 30, 1052–1071. doi: 10.1111/j.1365-3040.2007.01683.x 17661747

[B59] Olszewski J. Makowska M. Pszczolkowska A. Okorski A. Bieniaszewski T. (2014). The effect of nitrogen fertilization on flag leaf and ear photosynthesis and grain yield of spring wheat. Plant Soil Environ. 60, 531–536. doi: 10.17221/880/2013-Pse

[B60] Pallotti C. Renau-Morata B. Cardone L. Nebauer S. G. Palacios M. A. Rivas-Sendra A.. (2024). Understanding the saffron corm development-insights into histological and metabolic aspects. Plants 13. doi: 10.3390/plants13081125 PMC11055066 38674534

[B61] Panda D. Mohanty S. Das S. Mishra B. Baig M. J. Behera L. (2023). Light intensity-mediated auxin homeostasis in spikelets links carbohydrate metabolism enzymes with grain filling rate in rice. Protoplasma 260, 1233–1251. doi: 10.1007/s00709-023-01844-8 36847862

[B62] Paradiso R. Arena C. Rouphael Y. D’Aquino L. Makris K. Vitaglione P.. (2019). Growth, photosynthetic activity and tuber quality of two potato cultivars in controlled environment as affected by light source. Plant Biosys. 153, 725–735. doi: 10.1080/11263504.2018.1549603

[B63] Patro R. Duggal G. Love M. I. Irizarry R. A. Kingsford C. (2017). Salmon provides fast and bias-aware quantification of transcript expression. Nat. Methods 14, 417–419. doi: 10.1038/nmeth.4197 28263959 PMC5600148

[B64] Paul M. J. Foyer C. H. (2001). Sink regulation of photosynthesis. J. Exp. Bot. 52, 1383–1400. doi: 10.1093/jexbot/52.360.1383 11457898

[B65] Perrin P. M. Mitchell F. J. G. (2013). Effects of shade on growth, biomass allocation and leaf morphology in European yew (*Taxus baccata* L.). Eur. J. For. Res. 132, 211–218. doi: 10.1007/s10342-012-0668-8

[B66] Poorter H. Nagel O. (2000). The role of biomass allocation in the growth response of plants to different levels of light, CO2, nutrients and water: a quantitative review. Funct. Plant Biol. 27, 1191. doi: 10.1016/j.micromeso.2005.01.019

[B67] Poorter H. Niklas K. J. Reich P. B. Oleksyn J. Poot P. Mommer L. (2012). Biomass allocation to leaves, stems and roots: meta-analyses of interspecific variation and environmental control. New Phytol. 193, 30–50. doi: 10.1111/j.1469-8137.2011.03952.x 22085245

[B68] Porra R. J. Thompson W. A. Kriedemann P. E. (1989). Determination of accurate extinction coefficients and simultaneous equations for assaying chlorophylls a and b extracted with four different solvents: verification of the concentration of chlorophyll standards by atomic absorption spectroscopy. Biochim. Biophys. Acta -Bioener. 975, 384–394. doi: 10.1016/S0005-2728(89)80347-0

[B69] Quick W. P. Schaffer A. A. (2017). “Sucrose metabolism in sources and sinks,” in Photoassimilate Distribution in Plants and Crops Source-Sink Relationships, (New York: CRC Press), 115–158. doi: 10.1201/9780203743539-6

[B70] Renau-Morata B. Nebauer S. G. Sánchez M. Molina R. V. (2012). Effect of corm size, water stress and cultivation conditions on photosynthesis and biomass partitioning during the vegetative growth of saffron (*Curcus sativus* L.). Ind. Crops Prod. 39, 40–46. doi: 10.1016/j.indcrop.2012.02.009

[B71] Rikiishi K. Matsuura T. Ikeda Y. Maekawa M. (2015). Light inhibition of shoot regeneration is regulated by endogenous abscisic acid level in calli derived from immature barley embryos. PloS One 10. doi: 10.1371/journal.pone.0145242 PMC4682856 26670930

[B72] Rizzalli R. Villalobos F. Orgaz F. (2002). Radiation interception, radiation-use efficiency and dry matter partitioning in garlic (*Allium sativum* L.). Eur. J. Agron. 18, 33–43. doi: 10.1016/S1161-0301(02)00094-1

[B73] Robinson M. D. McCarthy D. J. Smyth G. K. (2010). edgeR: a Bioconductor package for differential expression analysis of digital gene expression data. Bioinformatics 26, 139–140. doi: 10.1093/bioinformatics/btp616 19910308 PMC2796818

[B74] Saldaña Villota T. M. Patiño J. A. Cotes-Torres J. M. (2015). Biomass distribution and allocation in diploid potato varieties (*Solanum phureja* Juz. et Buk.). Agronomía Colombiana. 33, 322–329. doi: 10.15446/agron.colomb.v33n3.50237

[B75] Schnyder H. (1993). The role of carbohydrate storage and redistribution in the source-sink relations of wheat and barley during grain filling—a review. New phytol. 123, 233–245. doi: 10.2307/2557991

[B76] Sepaskhah A. R. Amini-Nejad M. Kamgar-Haghighi A. A. (2013). Developing a dynamic yield and growth model for saffron under different irrigation regimes. Int. J. Plant Product. 7, 473–503. doi: 10.1016/j.abb.2005.07.011

[B77] Setter T. Ella E. Valdez A. (1994). Relationship between coleoptile elongation and alcoholic fermentation in rice exposed to anoxia. II. Cultivar differ. Ann. Bot. 74, 273–279. doi: 10.1006/anbo.1994.1118

[B78] Sharma S. Deswal R. (2021). *Dioscorea alata* tuber proteome analysis uncovers differentially regulated growth-associated pathways of tuber development. Plant Cell Physiol. 62, 191–204. doi: 10.1093/pcp/pcaa151 33313836

[B79] Sheikh F. R. Jose-Santhi J. Kalia D. Singh K. Singh R. K. (2022). Sugars as the regulators of dormancy and sprouting in geophytes. Ind. Crops Prod. 189. doi: 10.1016/j.indcrop.2022.115817

[B80] Shokrpour M. (2019). “Saffron (*Crocus sativus* L.) breeding: opportunities and challenges,” in Advances in Plant Breeding Strategies: Industrial and Food Crops, (Berlin: Springer International Publishing). vol. 6, 675–706. doi: 10.1007/978-3-030-23265-8_17

[B81] Smith M. R. Rao I. M. Merchant A. (2018). Source-sink relationships in crop plants and their influence on yield development and nutritional quality. Front. Plant Sci. 9. doi: 10.3389/fpls.2018.01889 PMC6306447 30619435

[B82] Smith A. M. Stitt M. (2007). Coordination of carbon supply and plant growth. Plant Cell Environ. 30, 1126–1149. doi: 10.1111/j.1365-3040.2007.01708.x 17661751

[B83] Taski-Ajdukovic K. Nagl N. Kovacev L. Curcic Z. Danojevic D. (2012). Development and application of qRT-PCR for sugar beet gene expression analysis in response to induced water deficit. Electron. J. Biotechnol. 15. doi: 10.2225/vol15-issue6-fulltext-9

[B84] Tavakkol-Afshari J. Brook A. Mousavi S. H. (2008). Study of cytotoxic and apoptogenic properties of saffron extract in human cancer cell lines. Food Chem. Toxicol. 46, 3443–3447. doi: 10.1016/j.fct.2008.08.018 18790714

[B85] Thompson A. J. Jackson A. C. Parker R. A. Morpeth D. R. Burbidge A. Taylor I. B. (2000). Abscisic acid biosynthesis in tomato: regulation of zeaxanthin epoxidase and 9-cis-epoxycarotenoid dioxygenase mRNAs by light/dark cycles, water stress and abscisic acid. Plant Mol. Biol. 42, 833–845. doi: 10.1023/a:1006448428401 10890531

[B86] Tivendale N. D. Millar A. H. (2022). How is auxin linked with cellular energy pathways to promote growth? New Phytol. 233, 2397–2404. doi: 10.1111/nph.17946 34984715

[B87] Turgeon R. (1989). The sink-source transition in leaves. Annu. Rev. Plant Biol. Plant Mol. Biol. 40, 119–138. doi: 10.1146/annurev.pp.40.060189.001003

[B88] van Doorn W. G. (2008). Is the onset of senescence in leaf cells of intact plants due to low or high sugar levels? J. Exp. Bot. 59, 1963–1972. doi: 10.1093/jxb/ern076 18453532

[B89] Vennapusa A. R. Somayanda I. M. Doherty C. J. Jagadish S. V. K. (2020). A universal method for high-quality RNA extraction from plant tissues rich in starch, proteins and fiber. Sci. Rep. 10, 16887. doi: 10.1038/s41598-020-73958-5 33037299 PMC7547072

[B90] Verdaguer D. Sala A. Vila M. (2010). Effect of environmental factors and bulb mass on the invasive geophyte *Oxalis pes-caprae* development. Acta Oecol. 36, 92–99. doi: 10.1016/j.actao.2009.10.006

[B91] Vollsnes A. V. Melo T. B. Futsaether C. M. (2012). Photomorphogenesis and pigment induction in lentil seedling roots exposed to low light conditions. Plant Biol. 14, 467–474. doi: 10.1111/j.1438-8677.2011.00516.x 22117590

[B92] Vreugdenhil D. Bindels P. Reinhoud P. Klocek J. Hendriks T. (1994). Use of the growth retardant tetcyclacis for potato tuber formation *in vitro* . Plant Growth Regul. 14, 257–265. doi: 10.1007/BF00024801

[B93] Wang Q. M. Hou F. Y. Dong S. X. Xie B. T. Li A. X. Zhang H. Y.. (2014). Effects of shading on the photosynthetic capacity, endogenous hormones and root yield in purple-fleshed sweetpotato (*lpomoea batatas* (L.) Lam). Plant Growth Regul. 72, 113–122. doi: 10.1007/s10725-013-9842-3

[B94] Wang Z. Li X. Xu J. X. Yang Z. Zhang Y. C. (2021). Effects of ambient temperature on flower initiation and flowering in saffron (*Crocus sativus* L.). Sci. Horticult. 279. doi: 10.1016/j.scienta.2020.109859

[B95] Xie C. Mao X. Z. Huang J. J. Ding Y. Wu J. M. Dong S.. (2011). KOBAS 2.0: a web server for annotation and identification of enriched pathways and diseases. Nucleic Acids Res. 39, W316–WW22. doi: 10.1093/nar/gkr483 21715386 PMC3125809

[B96] Xu M. L. Hu T. Q. Poethig R. S. (2021a). Low light intensity delays vegetative phase change. Plant Physiol. 187, 1177–1188. doi: 10.1093/plphys/kiab243 34618024 PMC8566249

[B97] Xu J. X. Li Q. Z. Li Y. Yang L. Y. Zhang Y. C. Cai Y. M. (2021b). Effect of exogenous gibberellin, paclobutrazol, abscisic acid, and ethrel application on bulblet development in. Front. Plant Sci. 11. doi: 10.3389/fpls.2020.615547 PMC7855306 33552107

[B98] Xu X. van Lammeren A. A. Vermeer E. Vreugdenhil D. (1998). The role of gibberellin, abscisic acid, and sucrose in the regulation of potato tuber formation *in vitro* . Plant Physiol. 117, 575–584. doi: 10.1104/pp.117.2.575 9625710 PMC34977

[B99] Xu K. Xu X. Fukao T. Canlas P. Maghirang-Rodriguez R. Heuer S.. (2006). Sub1A is an ethylene-response-factor-like gene that confers submergence tolerance to rice. Nature 442, 705–708. doi: 10.1038/nature04920 16900200

[B100] Yang F. Fan Y. F. Wu X. L. Cheng Y. J. Liu Q. L. Feng L. Y.. (2018). Auxin-to-gibberellin ratio as a signal for light intensity and quality in regulating soybean growth and matter partitioning. Front. Plant Sci. 9. doi: 10.3389/fpls.2018.00056 PMC5797538 29441084

[B101] Yang X. Zhao C. F. Zhou L. J. Wang Y. Liu X. H. (2016). Distinct impact of different types of aerosols on surface solar radiation in China. J. Geophys. Research-Atmos. 121, 6459–6471. doi: 10.1002/2016jd024938

[B102] Yao X. D. Li C. H. Li S. Y. Zhu Q. Zhang H. J. Wang H. Y.. (2017). Effect of shade on leaf photosynthetic capacity, light-intercepting, electron transfer and energy distribution of soybeans. Plant Growth Regul. 83, 409–416. doi: 10.1007/s10725-017-0307-y

[B103] Yu G. Mou Y. Shoaib N. He X. Liu L. Di R.. (2023a). Serine 31 Phosphorylation-driven regulation of AGPase activity: Potential implications for enhanced starch yields in crops. Int. J. Mol. Sci. 24. doi: 10.3390/ijms242015283 PMC10607544 37894964

[B104] Yu G. W. Shoaib N. Yang Y. Liu L. Mughal N. Mou Y. W.. (2023b). Effect of phosphorylation sites mutations on the subcellular localization and activity of AGPase Bt2 subunit: Implications for improved starch biosynthesis in maize. Agronomy 13. doi: 10.3390/agronomy13082119

[B105] Yu J. P. Xu S. J. Liu X. Y. Li T. Zhang D. H. Teng N. J.. (2022). Starch degradation and sucrose accumulation of lily bulbs after cold storage. Int. J. Mol. Sci. 23. doi: 10.3390/ijms23084366 PMC9029042 35457184

[B106] Yu X. R. Zhang J. Shao S. S. Yu H. Xiong F. Wang Z. (2015). Morphological and physicochemical properties of bulb and bulbil starches from. Starch-Stärke 67, 448–458. doi: 10.1002/star.201400209

[B107] Zhao N. Yu G. R. Wang Q. F. Wang R. L. Zhang J. H. Liu C. C.. (2020). Conservative allocation strategy of multiple nutrients among major plant organs: From species to community. J. Ecol. 108, 267–278. doi: 10.1111/1365-2745.13256

[B108] Zhou T. Chang F. Li X. Yang W. J. Huang X. L. Yan J.. (2024). Role of auxin and gibberellin under low light in enhancing saffron corm starch degradation during sprouting. Int. J. Biol. Macromol. 279. doi: 10.1016/j.ijbiomac.2024.135234 39218189

[B109] Zhou G. F. Li L. Q. Lu J. M. Li J. Yao C. Sun P.. (2020). Flower cultivation regimes affect apocarotenoid accumulation and gene expression during the development of saffron stigma. Horticult. Environ. Biotechnol. 61, 473–484. doi: 10.1007/s13580-020-00248-4

[B110] Zhou T. Qiu X. Zhao L. Yang W. J. Wen F. Y. Wu Q. H.. (2022). Optimal light intensity and quality increased the saffron daughter corm yield by inhibiting the degradation of reserves in mother corms during the reproductive stage. Ind. Crops Prod. 176. doi: 10.1016/j.indcrop.2021.114396

[B111] Zhou T. Wang L. Sun X. Wang X. C. Pu T. Yang H.. (2021). Improved post-silking light interception increases yield and P-use efficiency of maize in maize/soybean relay strip intercropping. Field Crop Res. 262. doi: 10.1016/j.fcr.2020.108054

[B112] Zhou T. Wang L. Yang H. Gao Y. Liu W. G. Yang W. Y. (2019). Ameliorated light conditions increase the P uptake capability of soybean in a relay-strip intercropping system by altering root morphology and physiology in the areas with low solar radiation. Sci. Total Environ. 688, 1069–1080. doi: 10.1016/j.scitotenv.2019.06.344 31726538

[B113] Zierer W. Rüscher D. Sonnewald U. Sonnewald S. (2021). Tuber and tuberous root development. Annu. Rev. Plant Biol. 72, 551–580. doi: 10.1146/annurev-arplant-080720-084456 33788583

