# Supplementary figures and images for: Low light reduces saffron corm yield by inhibiting starch synthesis
Source: Front Plant Sci. 2025 Jan 31;16:1544054. doi: 10.3389/fpls.2025.1544054 (PMC11825349; doi:10.3389/fpls.2025.1544054)

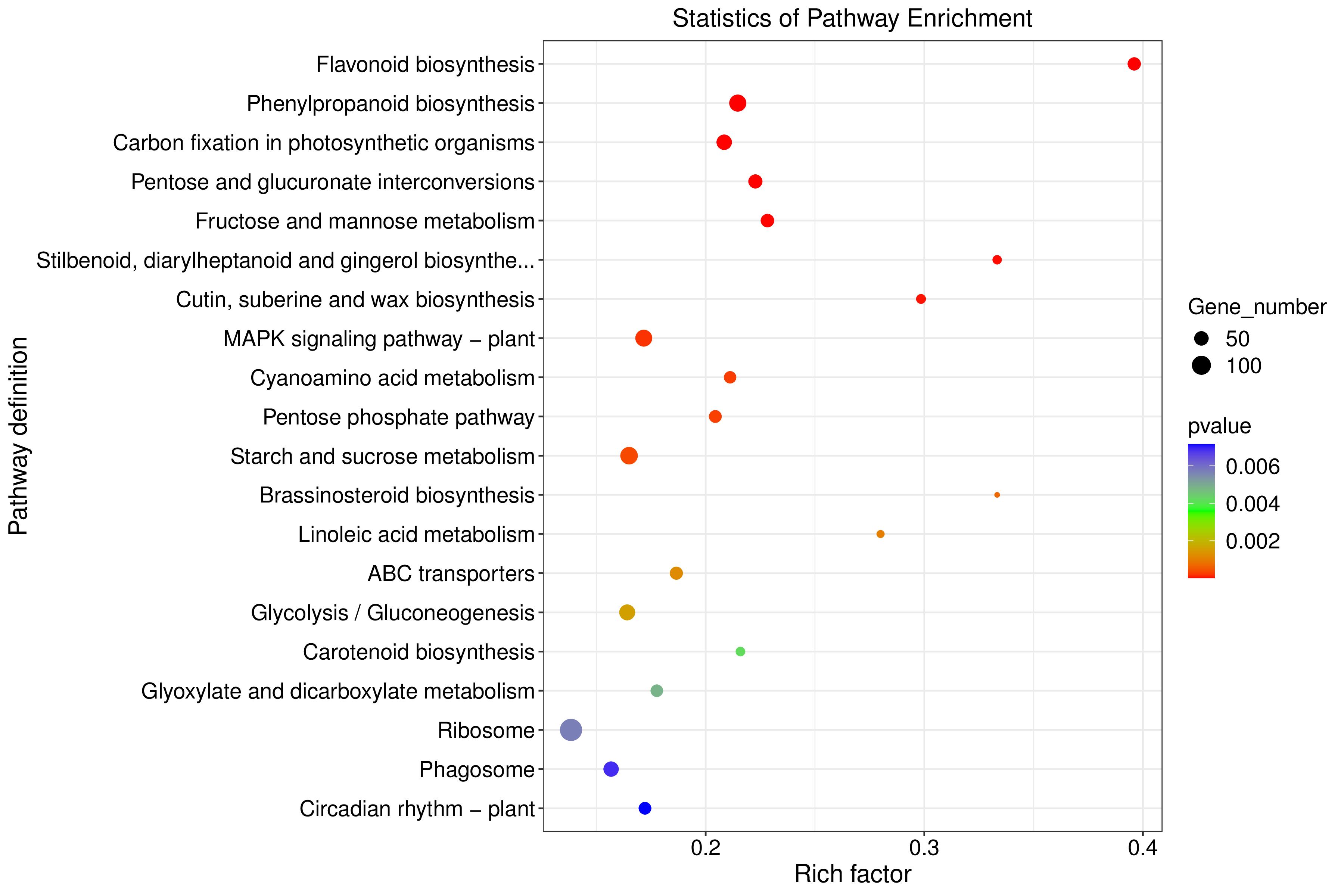

Supplement: Supplementary file 3 [file DataSheet3.zip › Raw Data and figures/Original figure/Figure 10/Figure 10A.jpg]

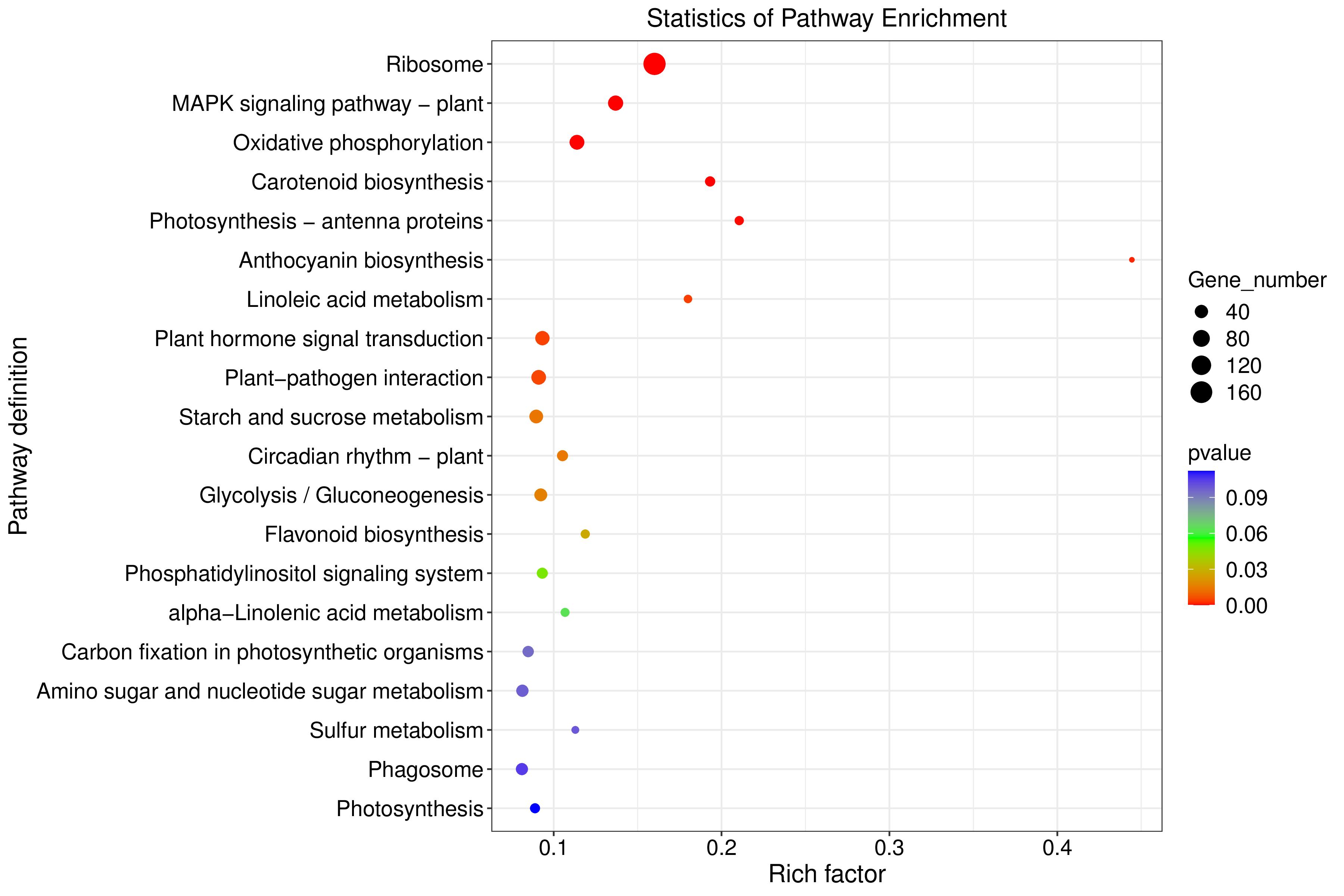

Supplement: Supplementary file 3 [file DataSheet3.zip › Raw Data and figures/Original figure/Figure 10/Figure 10B.jpg]

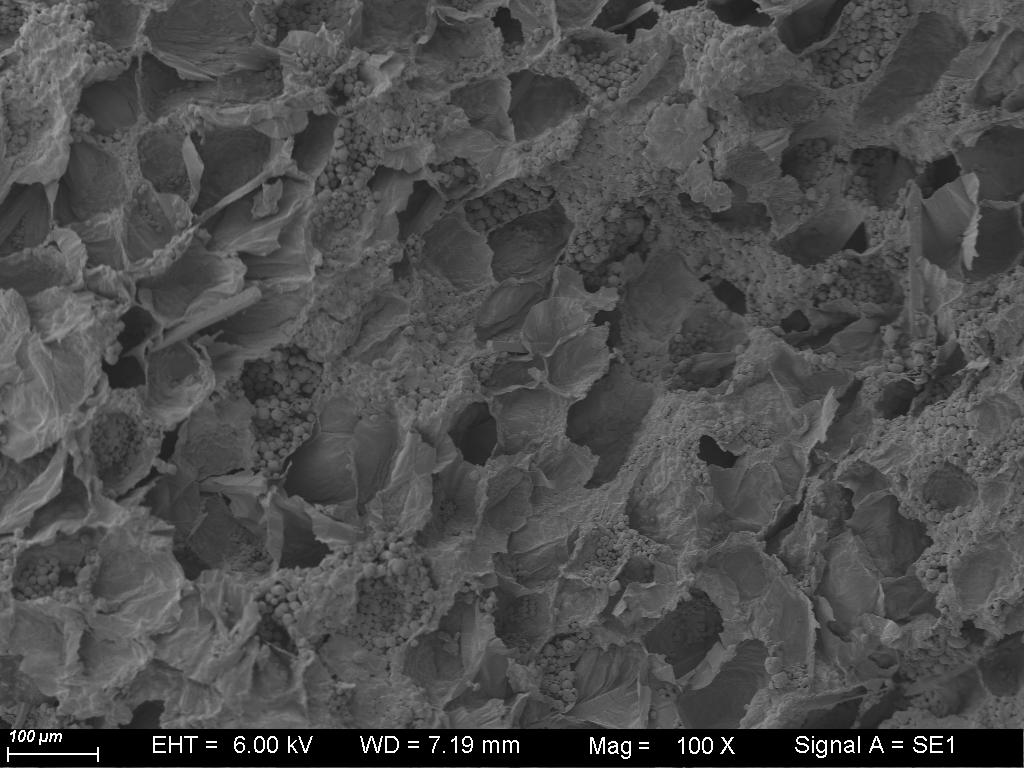

Supplement: Supplementary file 3 [file DataSheet3.zip › Raw Data and figures/Original figure/Figure 5/Figure 5E.jpg]

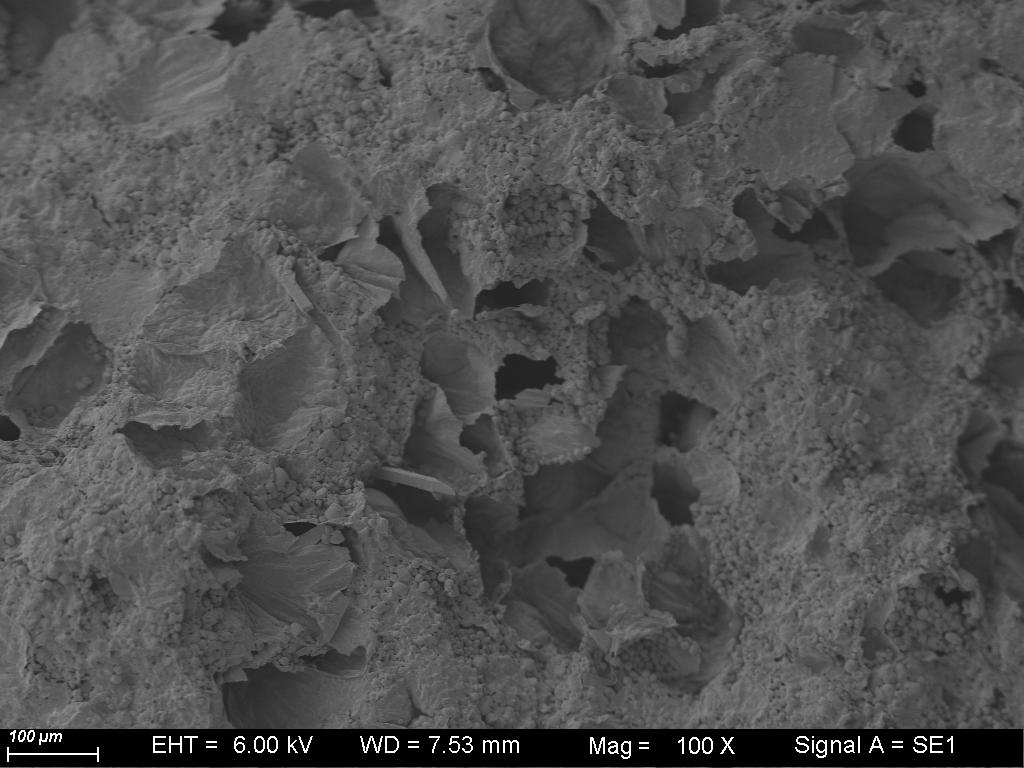

Supplement: Supplementary file 3 [file DataSheet3.zip › Raw Data and figures/Original figure/Figure 5/Figure 5F.jpg]

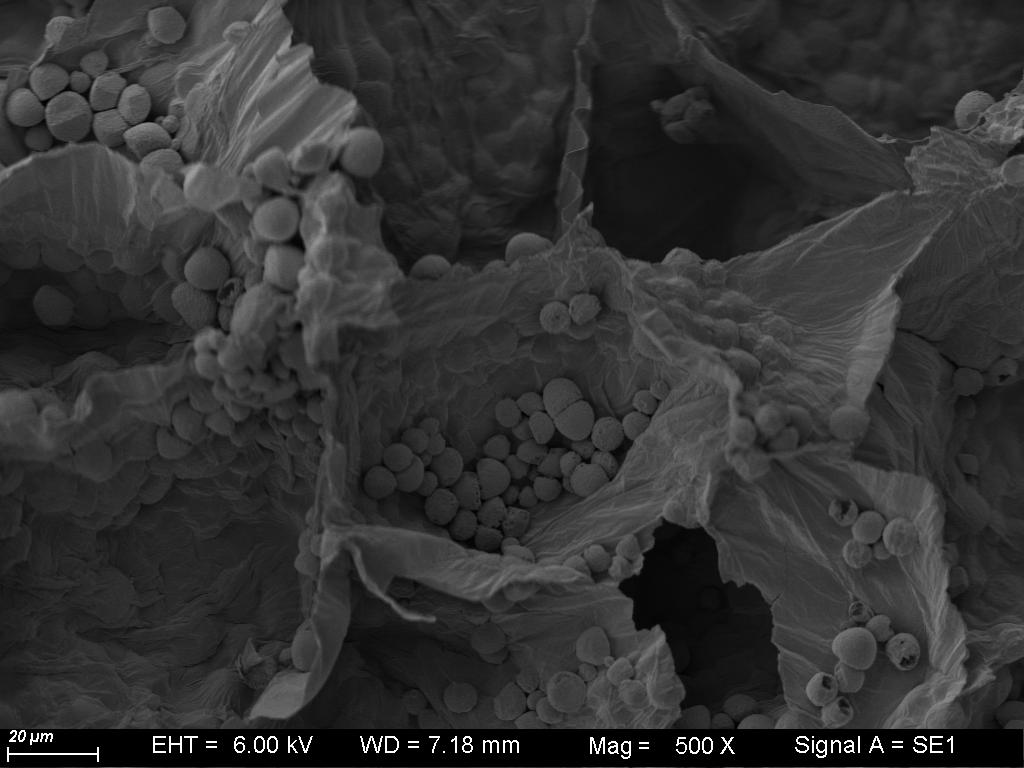

Supplement: Supplementary file 3 [file DataSheet3.zip › Raw Data and figures/Original figure/Figure 5/Figure 5G.jpg]

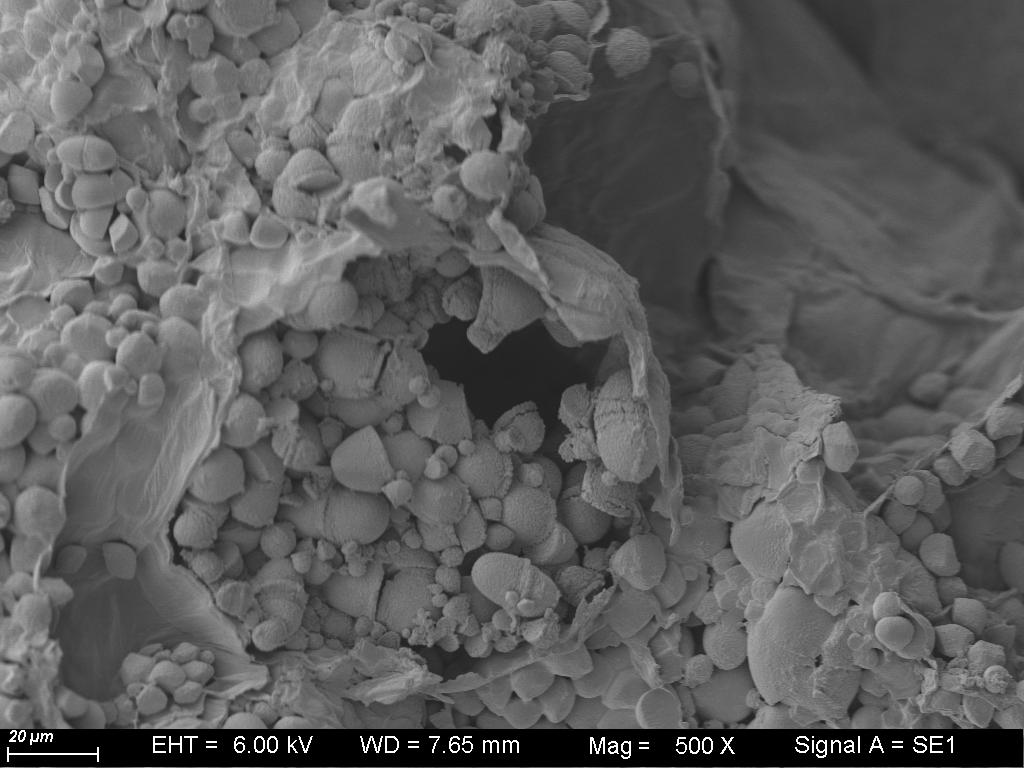

Supplement: Supplementary file 3 [file DataSheet3.zip › Raw Data and figures/Original figure/Figure 5/Figure 5H.jpg]

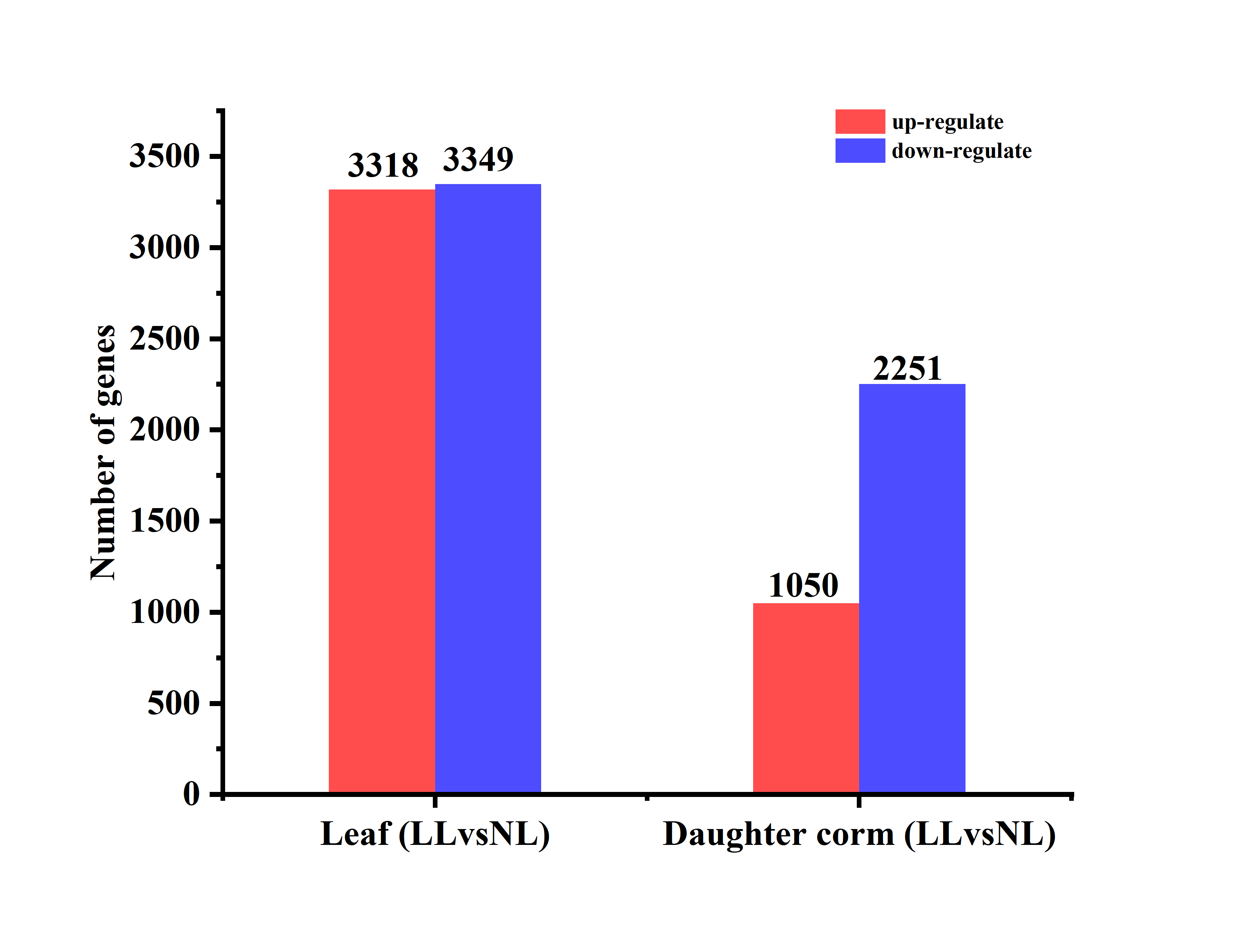

Supplement: Supplementary file 3 [file DataSheet3.zip › Raw Data and figures/Original figure/Supplementary Figure 7/Supplementary Figure 7A.jpg]

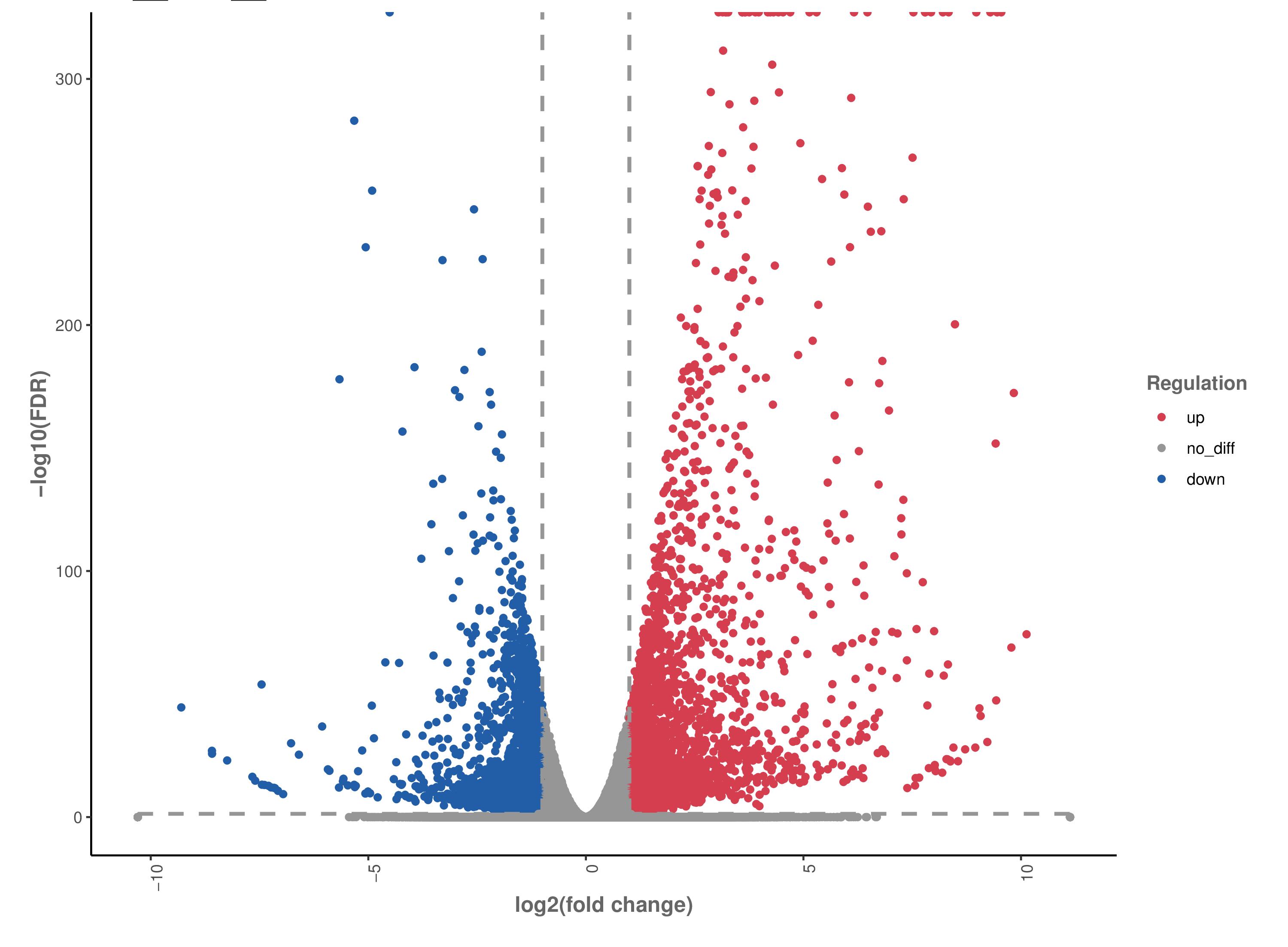

Supplement: Supplementary file 3 [file DataSheet3.zip › Raw Data and figures/Original figure/Supplementary Figure 7/Supplementary Figure 7B.jpg]

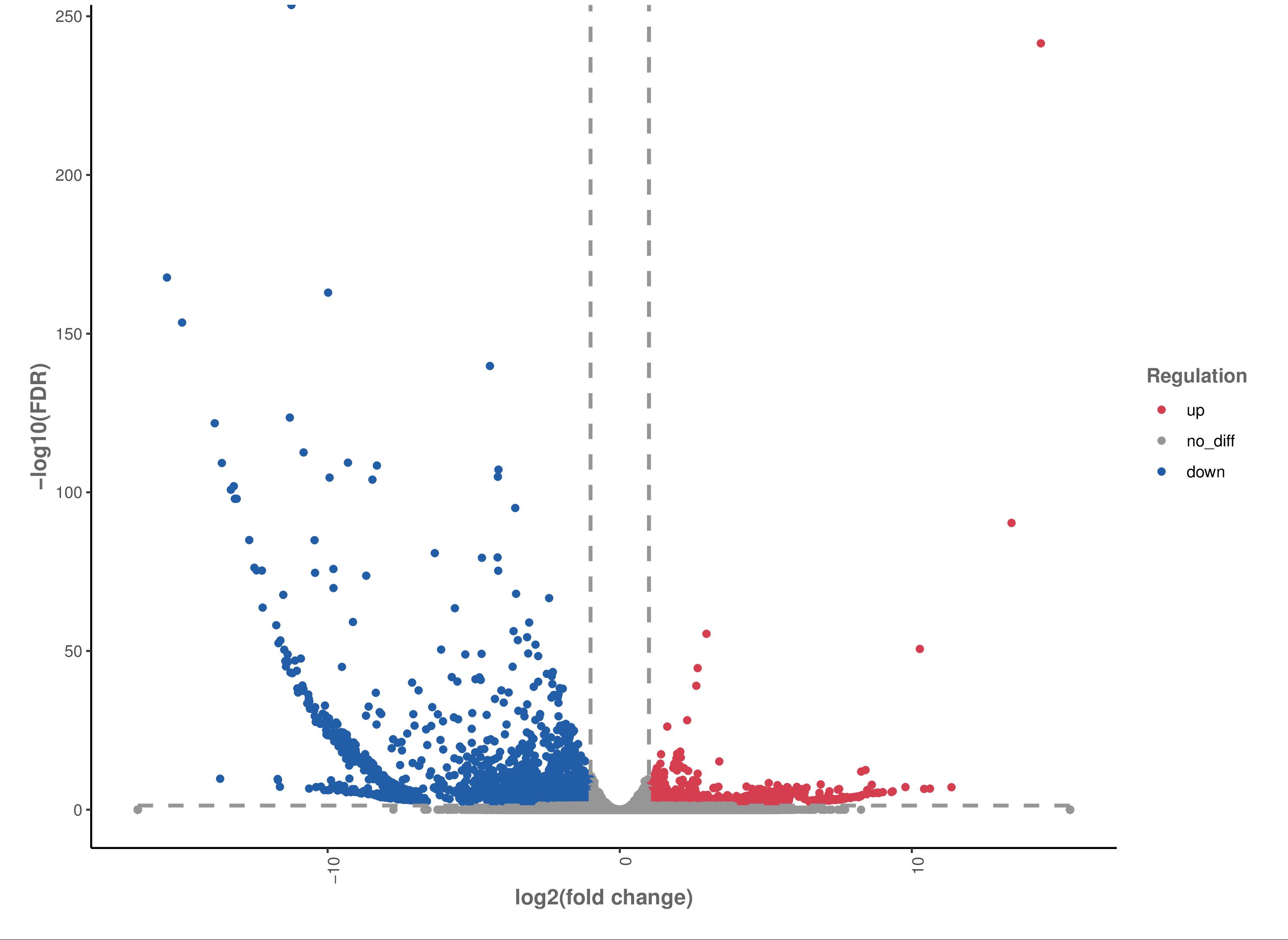

Supplement: Supplementary file 3 [file DataSheet3.zip › Raw Data and figures/Original figure/Supplementary Figure 7/Supplementary Figure 7C.jpg]
