# Supplementary material for: Low light reduces saffron corm yield by inhibiting starch synthesis
Source: Front Plant Sci. 2025 Jan 31;16:1544054. doi: 10.3389/fpls.2025.1544054 (PMC11825349; doi:10.3389/fpls.2025.1544054)
Supplement: Supplementary file 1 [file DataSheet1.zip › Supplemental Tables.doc]

**Supplementary Table 1 Primer sequence**

| Gene name | Forward primer sequence | Reverse primer sequence |
| --- | --- | --- |
| *CsINV1*  (TRINITY_DN27671_c0_g1) | ATTGGATACGGGCGAAGCAC | AGCAGGCAGACGACTGACCT |
| *CsHK1*  (TRINITY_DN22001_c0_g1) | TTAACACGGAATGGGGAGCC | CCGATACGCAGTCATCGTGA |
| *CsPFK2*  (TRINITY_DN95791_c0_g1) | CAGAGGCTGAAGCAGAACGG | TTCGCTGGAACTGCCCTAAC |
| *CsPK*  (TRINITY_DN30320_c0_g1) | CTGGGAAGCCTGCCATAAT | AGCACGAACCGCTGAAGAT |
| *CsSUS*  (TRINITY_DN29504_c0_g1) | TTCCACAAGACACTCCGT | TTCTGAGGAGCATCTCACTC |
| *CsAGPS1*  (TRINITY_DN41516_c0_g1) | TGGGAAGTGAAGGAAGTGGA | GTCTGAGTGGCTGCAAGGAC |

**Supplementary Table 2 The concentration of GA_1_, GA_3_, GA_4_ and GA_7_ in different organs**

| **Items** | **Treatment** | **Daughter Corm** | **Leaf** |
| --- | --- | --- | --- |
| **GA_1_** | **NL** | 0.338±0.005 | 3.14±0.58 |
|  | **LL** | 0.451±0.003*** | 3.76±0.14** |
| **GA_3_** | **NL** | - | 1.93±0.38 |
|  | **LL** | - | 2.83±0.04 |
| **GA_4_** | **NL** | 0.37±0.03 | - |
|  | **LL** | 0.33±0.01 | - |
| **GA_7_** | **NL** | - | - |
|  | **LL** | - | - |

The experiments conducted in 2022~2023, and the plants were grown under low light intensity (LL) and natural light intensity (NL). Samples were manipulated in March of the following year. All data were the mean ± standard error of three replicates. GA, Gibberellin. Significant differences between LL and NL treatments were determined using Student's t-test, ***p*≤0.01, ****p*≤0.001.“-” means no detected.

**Supplementary Table 3 List of glycolysis pathway genes in leaves analysed by KEGG enrichment**

| Gene_ID | log_2_FC | Name | Annotation |
| --- | --- | --- | --- |
| TRINITY_DN2911_c0_g1 | 1.19 | ALDH3F1 | aldehyde dehydrogenase family 3 member F1-like [Asparagus officinalis] |
| TRINITY_DN29331_c0_g1 | -1.17 | ALDP | fructose-bisphosphate aldolase, chloroplastic [Asparagus officinalis] |
| TRINITY_DN30028_c0_g1 | -1.20 | FBPban1 | hypothetical protein PRUPE_8G028300 [Prunus persica] |
| TRINITY_DN30320_c0_g1 | 1.01 | PK | pyruvate kinase 1, cytosolic [Asparagus officinalis] |
| TRINITY_DN30333_c0_g1 | -1.82 | ALDP | fructose-bisphosphate aldolase 1, chloroplastic-like [Olea europaea var. sylvestris] |
| TRINITY_DN30576_c0_g1 | -1.28 | ALDP | fructose-1,6-diphosphate aldolase [Metasequoia glyptostroboides] |
| TRINITY_DN320_c0_g1 | -1.39 | ALDP | hypothetical protein DCAR_013264 [Daucus carota subsp. sativus] |
| TRINITY_DN320_c0_g2 | 1.11 | FBA1 | LOW QUALITY PROTEIN: fructose-bisphosphate aldolase 1, cytoplasmic-like [Asparagus officinalis] |
| TRINITY_DN33528_c0_g1 | -1.14 | ALDP | unknown [Picea sitchensis] |
| TRINITY_DN34232_c0_g1 | 1.10 | Fbp1 | Fructose-1,6-bisphosphatase class 1/Sedoheputulose-1,7-bisphosphatase [Corchorus capsularis] |
| TRINITY_DN35188_c0_g1 | -1.18 | ALDP | fructose-bisphosphate aldolase 1, chloroplastic [Ziziphus jujuba] |
| TRINITY_DN37738_c0_g1 | -1.47 | FBPban1 | hypothetical protein C5167_017458 [Papaver somniferum] |
| TRINITY_DN40427_c0_g1 | -1.30 | FBPban1 | - |
| TRINITY_DN4214_c0_g1 | -1.14 | ALDP | fructose-bisphosphate aldolase, chloroplastic-like [Phoenix dactylifera] |
| TRINITY_DN43736_c1_g1 | -1.67 | FBPban1 | fructose-1,6-bisphosphatase, cytosolic isoform X1 [Aegilops tauschii subsp. tauschii] |
| TRINITY_DN68275_c0_g1 | 1.82 | PK | PREDICTED: pyruvate kinase 1, cytosolic [Elaeis guineensis] |
| TRINITY_DN7610_c0_g1 | -1.09 | PGK | PREDICTED: phosphoglycerate kinase, chloroplastic-like [Elaeis guineensis] |
| TRINITY_DN762_c0_g1 | 2.85 | GALM | aldose 1-epimerase [Asparagus officinalis] |
| TRINITY_DN789_c0_g1 | -1.01 | FBPban1 | PREDICTED: fructose-1,6-bisphosphatase, cytosolic-like [Elaeis guineensis] |
| TRINITY_DN82330_c0_g1 | 1.15 | Akr1c13 | PREDICTED: non-functional NADPH-dependent codeinone reductase 2-like [Fragaria vesca subsp. vesca] |
| TRINITY_DN8295_c0_g1 | -1.00 | G6P-1-E | uncharacterized protein A4U43_C01F36120 [Asparagus officinalis] |
| TRINITY_DN8581_c0_g2 | 1.10 | PK | PREDICTED: pyruvate kinase, cytosolic isozyme [Vitis vinifera] |
| TRINITY_DN8802_c0_g1 | 1.14 | PK | pyruvate kinase, cytosolic isozyme [Asparagus officinalis] |
| TRINITY_DN8879_c0_g1 | 1.48 | PDHB-1 | PREDICTED: pyruvate dehydrogenase E1 component subunit beta-1, mitochondrial [Musa acuminata subsp. malaccensis] |
| TRINITY_DN15398_c0_g1 | 1.01 | mipp1 | multiple inositol polyphosphate phosphatase 1-like [Asparagus officinalis] |
| TRINITY_DN16250_c0_g1 | 2.21 | GALM | aldose 1-epimerase [Asparagus officinalis] |
| TRINITY_DN17004_c0_g1 | -1.08 | GAPCP1 | glyceraldehyde-3-phosphate dehydrogenase [Ornithogalum longebracteatum] |
| TRINITY_DN18238_c0_g1 | -1.51 | FBPban1 | hypothetical protein B296_00051537 [Ensete ventricosum] |
| TRINITY_DN18238_c0_g2 | -1.20 | FBPban1 | fructose-1,6-bisphosphatase cytosolic-like, partial [Trifolium pratense] |
| TRINITY_DN19225_c0_g1 | 4.08 | GALM | hypothetical protein GW17_00025762 [Ensete ventricosum] |
| TRINITY_DN19513_c0_g1 | -1.01 | PNSB2 | photosynthetic NDH subunit of subcomplex B 2, chloroplastic [Asparagus officinalis] |
| TRINITY_DN1971_c3_g1 | -1.06 | ALDP | PREDICTED: fructose-bisphosphate aldolase, chloroplastic [Elaeis guineensis] |
| TRINITY_DN22001_c0_g1 | 1.27 | Hk1 | hexokinase [Artemisia annua] |
| TRINITY_DN23396_c0_g1 | 1.43 | ALDH3H1 | aldehyde dehydrogenase [Crocus sativus] |
| TRINITY_DN23676_c0_g1 | 1.01 | PK | uncharacterized protein A4U43_C07F21470 [Asparagus officinalis] |
| TRINITY_DN2380_c0_g1 | -1.06 | ALDP | fructose-bisphosphate aldolase, chloroplastic-like [Phoenix dactylifera] |
| TRINITY_DN24296_c2_g1 | -1.88 | ALDP | hypothetical protein B296_00046326 [Ensete ventricosum] |
| TRINITY_DN24482_c0_g1 | -1.10 | ALDP | fructose-bisphosphate aldolase, chloroplastic [Phoenix dactylifera] |
| TRINITY_DN26020_c0_g1 | 1.27 | TPI | triosephosphate isomerase, cytosolic [Phoenix dactylifera] |
| TRINITY_DN26940_c0_g1 | -1.34 | GAPC2 | hypothetical protein B456_012G089500 [Gossypium raimondii] |
| TRINITY_DN2715_c0_g1 | 1.05 | FBA1 | LOW QUALITY PROTEIN: fructose-bisphosphate aldolase 1, cytoplasmic-like [Asparagus officinalis] |
| TRINITY_DN9493_c1_g2 | -1.45 | PFP-BETA | PREDICTED: pyrophosphate--fructose 6-phosphate 1-phosphotransferase subunit beta-like [Elaeis guineensis] |
| TRINITY_DN95359_c0_g2 | 1.28 | Akr1b1 | NADPH-dependent aldo-keto reductase, chloroplastic-like [Cucurbita moschata] |
| TRINITY_DN95791_c0_g1 | 2.67 | PFK2 | PREDICTED: ATP-dependent 6-phosphofructokinase 2-like [Elaeis guineensis] |
| TRINITY_DN4664_c1_g1 | -1.14 | PGK | phosphoglycerate kinase, cytosolic-like [Phoenix dactylifera] |
| TRINITY_DN4818_c0_g1 | 1.17 | PK | plastidial pyruvate kinase 2 [Asparagus officinalis] |
| TRINITY_DN50348_c0_g1 | -1.19 | ALDP | PREDICTED: fructose-bisphosphate aldolase, chloroplastic [Elaeis guineensis] |
| TRINITY_DN5098_c1_g1 | 2.63 | ERG1 | elicitor-responsive protein 1 isoform X2 [Asparagus officinalis] |
| TRINITY_DN50_c1_g1 | -1.19 | PGK | phosphoglycerate kinase, chloroplastic [Asparagus officinalis] |
| TRINITY_DN52863_c0_g1 | 1.54 | FBA1 | PREDICTED: fructose-bisphosphate aldolase 1, cytoplasmic [Elaeis guineensis] |
| TRINITY_DN52945_c0_g1 | 1.07 | Pgam1 | unknown [Zea mays] |
| TRINITY_DN62701_c0_g2 | 1.66 | AP | alkaline phytase isoform 2 [Lilium longiflorum] |
| TRINITY_DN10048_c0_g1 | -1.65 | TPI | hypothetical protein C2845_PM07G28160 [Panicum miliaceum] |
| TRINITY_DN10486_c0_g1 | -1.03 | ALDP | PREDICTED: fructose-bisphosphate aldolase, chloroplastic [Musa acuminata subsp. malaccensis] |
| TRINITY_DN10908_c0_g1 | 4.76 | GALM | aldose 1-epimerase [Asparagus officinalis] |
| TRINITY_DN112588_c0_g1 | -1.23 | FBA2 | unnamed protein product [Brassica oleracea] |
| TRINITY_DN11380_c0_g1 | 1.12 | ADH | alcohol dehydrogenase class-3-like [Manihot esculenta] |
| TRINITY_DN11407_c0_g1 | -2.05 | TPI | triose phosphate isomerase3 [Zea mays] |
| TRINITY_DN117219_c0_g1 | 1.49 | Ldhc | PREDICTED: L-lactate dehydrogenase B [Tarenaya hassleriana] |
| TRINITY_DN119895_c0_g1 | 1.57 | Gapdhs | glyceraldehyde-3-phosphate dehydrogenase GAPCP1, chloroplastic [Amborella trichopoda] |
| TRINITY_DN1247_c0_g1 | 1.05 | PK | PREDICTED: pyruvate kinase, cytosolic isozyme [Elaeis guineensis] |
| TRINITY_DN13137_c0_g1 | -1.15 | FBPban1 | fructose-1,6-bisphosphatase, chloroplastic [Phoenix dactylifera] |
| TRINITY_DN13943_c0_g1 | -1.43 | FBA2 | fructose-bisphosphate aldolase, chloroplastic-like [Phoenix dactylifera] |

**Supplementary Table 4 List of starch and sucrose metabolism pathway genes in leaves analysed by KEGG enrichment**

| Gene_ID | log_2_FC | Name | Annotation |
| --- | --- | --- | --- |
| TRINITY_DN13943_c0_g1 | -1.43 | FBA2 | fructose-bisphosphate aldolase, chloroplastic-like [Phoenix dactylifera] |
| TRINITY_DN29504_c0_g1 | 1.04 | SUS | sucrose synthase [Ornithogalum longebracteatum] |
| TRINITY_DN2965_c0_g1 | 1.10 | SUS | sucrose synthase [Ornithogalum longebracteatum] |
| TRINITY_DN298_c0_g2 | 1.79 | GLU2 | endoglucanase 10-like [Asparagus officinalis] |
| TRINITY_DN31162_c0_g1 | -1.76 | LOC103997551 | PREDICTED: uncharacterized protein LOC103997551 isoform X1 [Musa acuminata subsp. malaccensis] |
| TRINITY_DN34917_c0_g1 | -1.32 | At5g44360 | berberine bridge enzyme-like 22 [Asparagus officinalis] |
| TRINITY_DN34970_c0_g1 | 2.35 | BGLU12 | beta-glucosidase 12-like isoform X3 [Asparagus officinalis] |
| TRINITY_DN35808_c0_g1 | 1.83 | BBE1 | reticuline oxidase-like [Phoenix dactylifera] |
| TRINITY_DN36741_c0_g1 | 3.66 | BGLU12 | beta-glucosidase 12-like [Asparagus officinalis] |
| TRINITY_DN36741_c0_g2 | 2.54 | BGLU12 | PREDICTED: beta-glucosidase 12-like isoform X1 [Elaeis guineensis] |
| TRINITY_DN37908_c0_g1 | 2.23 | TRE1 | probable trehalase isoform X1 [Phoenix dactylifera] |
| TRINITY_DN37908_c0_g2 | 2.52 | TRE2 | probable trehalase isoform X2 [Phoenix dactylifera] |
| TRINITY_DN38394_c0_g1 | 5.22 | SUS | sucrose synthase [Lilium davidii var. unicolor] |
| TRINITY_DN4238_c0_g2 | 1.63 | PGIP1 | PREDICTED: polygalacturonase inhibitor-like [Elaeis guineensis] |
| TRINITY_DN65337_c0_g1 | -1.58 | EGLC | PREDICTED: putative glucan endo-1,3-beta-glucosidase GVI [Elaeis guineensis] |
| TRINITY_DN6812_c0_g1 | 5.93 | SUS | uncharacterized protein A4U43_C06F3510 [Asparagus officinalis] |
| TRINITY_DN6893_c0_g1 | 1.42 | BGLU18 | beta-glucosidase 18-like [Ananas comosus] |
| TRINITY_DN69733_c0_g1 | -1.43 | LOC109823264 | uncharacterized protein LOC109823264 [Asparagus officinalis] |
| TRINITY_DN70584_c0_g1 | -1.28 | PCMP-E95 | PREDICTED: pentatricopeptide repeat-containing protein At3g22150, chloroplastic [Elaeis guineensis] |
| TRINITY_DN7130_c0_g1 | 1.60 | SUCS | sucrose synthase, partial [Balsamocarpon brevifolium] |
| TRINITY_DN7207_c0_g1 | -1.30 | EGLC | glucan endo-1,3-beta-glucosidase 10-like [Phoenix dactylifera] |
| TRINITY_DN7266_c0_g1 | 3.98 | EG6 | PREDICTED: endoglucanase 6 [Elaeis guineensis] |
| TRINITY_DN72968_c0_g1 | -1.24 | WAXY | granule-bound starch synthase 1b, chloroplastic/amyloplastic isoform X2 [Asparagus officinalis] |
| TRINITY_DN7574_c0_g2 | 1.84 | TPS9 | uncharacterized protein A4U43_C05F25470 [Asparagus officinalis] |
| TRINITY_DN8264_c0_g1 | 1.85 | LOC110026450 | uncharacterized protein LOC110026450 isoform X1 [Phalaenopsis equestris] |
| TRINITY_DN8273_c0_g1 | -1.29 | PCMP-H42 | PREDICTED: putative pentatricopeptide repeat-containing protein At3g47840 [Elaeis guineensis] |
| TRINITY_DN833_c1_g1 | 3.67 | EGLC | putative glucan endo-1,3-beta-glucosidase GVI [Phoenix dactylifera] |
| TRINITY_DN8519_c0_g1 | 2.76 | BACOVA_02659 | uncharacterized protein LOC109843897 [Asparagus officinalis] |
| TRINITY_DN8681_c0_g1 | -1.18 | WAXY | granule-bound starch synthase 1, chloroplastic/amyloplastic-like [Ananas comosus] |
| TRINITY_DN8851_c0_g1 | -1.48 | BGLU12 | beta-glucosidase 12-like [Asparagus officinalis] |
| TRINITY_DN15896_c0_g1 | -1.19 | EGLC | PREDICTED: glucan endo-1,3-beta-glucosidase 11-like [Elaeis guineensis] |
| TRINITY_DN16090_c0_g1 | 1.01 | GHF | glycosyl hydrolase 5 family protein-like [Phoenix dactylifera] |
| TRINITY_DN16173_c0_g1 | 3.14 | BGLU15 | putative inactive beta-glucosidase 14 [Ananas comosus] |
| TRINITY_DN16689_c0_g1 | 1.46 | BetaGluc1 | PREDICTED: uncharacterized protein LOC105042391 [Elaeis guineensis] |
| TRINITY_DN18323_c0_g3 | 1.58 | At3g47200 | UPF0481 protein [Apostasia shenzhenica] |
| TRINITY_DN18694_c0_g1 | -1.25 | BGLU11 | hypothetical protein [Delphinium grandiflorum] |
| TRINITY_DN19508_c0_g1 | 2.29 | SUS | sucrose synthase 2-like [Asparagus officinalis] |
| TRINITY_DN2054_c0_g1 | 3.50 | EG6 | PREDICTED: endoglucanase 6 [Elaeis guineensis] |
| TRINITY_DN21445_c0_g1 | 1.74 | EG17 | endoglucanase 17 [Cinnamomum micranthum f. kanehirae] |
| TRINITY_DN21751_c0_g1 | 4.94 | PGIP | PREDICTED: polygalacturonase inhibitor-like [Elaeis guineensis] |
| TRINITY_DN22001_c0_g1 | 1.27 | Hk1 | hexokinase [Artemisia annua] |
| TRINITY_DN23840_c0_g1 | 3.15 | BGLU18 | PREDICTED: beta-glucosidase 18-like [Musa acuminata subsp. malaccensis] |
| TRINITY_DN23840_c0_g2 | 1.82 | BGLU18 | PREDICTED: beta-glucosidase 18 isoform X1 [Elaeis guineensis] |
| TRINITY_DN27666_c0_g1 | -1.65 | TPPH | probable trehalose-phosphate phosphatase J isoform X1 [Durio zibethinus] |
| TRINITY_DN27671_c0_g1 | 1.87 | INV1 | cell wall invertase INV11 [Camellia sinensis] |
| TRINITY_DN28231_c0_g1 | 1.72 | GLU1 | PREDICTED: endoglucanase 10-like [Musa acuminata subsp. malaccensis] |
| TRINITY_DN28618_c0_g1 | -1.24 | LOC113279510 | uncharacterized protein LOC113279510 [Papaver somniferum] |
| TRINITY_DN2891_c1_g1 | -1.03 | EGLA | glucan endo-1,3-beta-glucosidase 14 [Asparagus officinalis] |
| TRINITY_DN89383_c0_g1 | 3.03 | PGIP1 | PREDICTED: polygalacturonase inhibitor-like [Musa acuminata subsp. malaccensis] |
| TRINITY_DN899_c0_g1 | 1.63 | GLU1 | PREDICTED: endoglucanase 10-like [Musa acuminata subsp. malaccensis] |
| TRINITY_DN899_c1_g2 | 1.89 | GLU3 | PREDICTED: endoglucanase 12-like [Elaeis guineensis] |
| TRINITY_DN90021_c0_g1 | 1.02 | Ugp2 | PREDICTED: UTP--glucose-1-phosphate uridylyltransferase 2-like [Tarenaya hassleriana] |
| TRINITY_DN914_c0_g1 | -1.58 | AGPB1 | glucose-1-phosphate adenylyltransferase small subunit, chloroplastic isoform X1 [Arabidopsis lyrata subsp. lyrata] |
| TRINITY_DN9248_c0_g1 | -2.00 | BAM1 | beta-amylase 1, chloroplastic [Phoenix dactylifera] |
| TRINITY_DN94412_c0_g1 | -1.35 | BGLU2 | hypothetical protein GQ55_5G117600 [Panicum hallii var. hallii] |
| TRINITY_DN9525_c0_g1 | 3.65 | EGLC | putative glucan endo-1,3-beta-glucosidase GVI [Phoenix dactylifera] |
| TRINITY_DN9775_c0_g1 | 2.11 | TPS9 | LOW QUALITY PROTEIN: probable alpha,alpha-trehalose-phosphate synthase [UDP-forming] 9 [Asparagus officinalis] |
| TRINITY_DN45_c0_g1 | 1.45 | SUS | sucrose synthase [Ornithogalum longebracteatum] |
| TRINITY_DN466_c0_g1 | -1.72 | WAXY | granule-bound starch synthase 1b, chloroplastic/amyloplastic isoform X2 [Asparagus officinalis] |
| TRINITY_DN47882_c0_g1 | 1.45 | INV1 | cell wall invertase [Agave tequilana] |
| TRINITY_DN4963_c0_g1 | 1.55 | EGLC | putative glucan endo-1,3-beta-glucosidase GVI [Asparagus officinalis] |
| TRINITY_DN5065_c1_g1 | -1.28 | TPPH | probable trehalose-phosphate phosphatase J [Coffea arabica] |
| TRINITY_DN5440_c0_g1 | -1.16 | ADG2 | glucose-1-phosphate adenylyltransferase large subunit 3, chloroplastic/amyloplastic isoform X1 [Asparagus officinalis] |
| TRINITY_DN5670_c0_g1 | 3.14 | GLU1 | LOW QUALITY PROTEIN: endoglucanase 9-like [Ananas comosus] |
| TRINITY_DN6440_c0_g2 | 1.33 | At3g49720 | uncharacterized protein At3g49720-like [Asparagus officinalis] |
| TRINITY_DN10682_c0_g2 | 2.93 | BACOVA_02659 | Beta-glucosidase BoGH3B [Ananas comosus] |
| TRINITY_DN10938_c0_g1 | 2.43 | BBE18 | berberine bridge enzyme-like 18 [Phoenix dactylifera] |
| TRINITY_DN10938_c0_g2 | -1.46 | BBE22 | berberine bridge enzyme-like 22 [Asparagus officinalis] |
| TRINITY_DN11602_c0_g1 | 9.84 | BG | basic 7S globulin-like [Phoenix dactylifera] |
| TRINITY_DN11637_c0_g1 | 1.06 | EGLC | glucan endo-1,3-beta-glucosidase 11-like [Phalaenopsis equestris] |
| TRINITY_DN1167_c0_g1 | 1.00 | EGLC | glucan endo-1,3-beta-glucosidase 7 [Phoenix dactylifera] |
| TRINITY_DN1167_c0_g2 | 1.37 | EGLC | PREDICTED: glucan endo-1,3-beta-glucosidase 7 [Elaeis guineensis] |
| TRINITY_DN11801_c1_g1 | -1.02 | GLU1 | uncharacterized protein LOC100501706 [Zea mays] |
| TRINITY_DN12006_c0_g1 | 1.94 | EGLC-A6 | probable glucan endo-1,3-beta-glucosidase A6 [Phoenix dactylifera] |
| TRINITY_DN1277_c0_g1 | 1.33 | BGLU12 | beta-glucosidase 12 [Crocus sativus] |
| TRINITY_DN12800_c0_g1 | 3.28 | SUS | uncharacterized protein A4U43_C06F3510 [Asparagus officinalis] |
| TRINITY_DN13649_c0_g1 | -1.78 | WAXY | granule-bound starch synthase 1, chloroplastic/amyloplastic [Phoenix dactylifera] |
| TRINITY_DN14337_c0_g1 | -1.21 | BGLU11 | uncharacterized protein LOC103716408 [Phoenix dactylifera] |
| TRINITY_DN1491_c0_g1 | 1.54 | At3g49720 | PREDICTED: uncharacterized protein At3g49720 isoform X1 [Elaeis guineensis] |
| TRINITY_DN1491_c0_g2 | 1.38 | At3g49720 | uncharacterized protein At3g49720-like [Asparagus officinalis] |

**Supplementary Table 5 List of starch and sucrose metabolism pathway genes in daughter corms analysed by KEGG enrichment**

| Gene_ID | log_2_FC | Name | Annotation |
| --- | --- | --- | --- |
| TRINITY_DN36092_c0_g2 | -7.36 | GPI | glucose-6-phosphate isomerase-like [Quercus suber] |
| TRINITY_DN41258_c0_g1 | -3.21 | PGM1 | hypothetical protein AALP_AA2G138100 [Arabis alpina] |
| TRINITY_DN41516_c0_g1 | 1.37 | AGPS1 | AGPL1 protein [Gladiolus hybrid cultivar] |
| TRINITY_DN41622_c0_g1 | -3.08 | BAM3 | PREDICTED: beta-amylase 3, chloroplastic [Elaeis guineensis] |
| TRINITY_DN4238_c0_g2 | -3.07 | PGIP1 | PREDICTED: polygalacturonase inhibitor-like [Elaeis guineensis] |
| TRINITY_DN66404_c0_g1 | 8.03 | BGLU23 | Glycosyl hydrolase superfamily protein [Arabidopsis thaliana] |
| TRINITY_DN70167_c0_g1 | 1.68 | WAXY | hypothetical protein B296_00020034 [Ensete ventricosum] |
| TRINITY_DN71389_c0_g3 | -9.70 | PYGM | glycosyltransferase family 35 protein [Micromonas commoda] |
| TRINITY_DN7213_c0_g1 | -4.72 | PGIP1 | PREDICTED: polygalacturonase inhibitor-like [Elaeis guineensis] |
| TRINITY_DN7574_c0_g2 | -1.14 | TPS9 | uncharacterized protein A4U43_C05F25470 [Asparagus officinalis] |
| TRINITY_DN7592_c0_g1 | -1.07 | SUS | SuSy protein [Gladiolus hybrid cultivar] |
| TRINITY_DN8051_c0_g1 | -1.20 | BBE1 | PREDICTED: reticuline oxidase-like [Musa acuminata subsp. malaccensis] |
| TRINITY_DN8264_c0_g1 | -3.16 | LOC110026450 | uncharacterized protein LOC110026450 isoform X1 [Phalaenopsis equestris] |
| TRINITY_DN15511_c0_g1 | -1.38 | exgA | probable glucan 1,3-beta-glucosidase A isoform X1 [Phoenix dactylifera] |
| TRINITY_DN16575_c0_g1 | -3.97 | IQM4 | IQ domain-containing protein IQM1-like [Dendrobium catenatum] |
| TRINITY_DN1681_c0_g1 | -2.42 | BAM1 | beta-amylase 1, chloroplastic [Phoenix dactylifera] |
| TRINITY_DN1681_c1_g1 | -2.73 | BAM3 | beta-amylase 3, chloroplastic [Phoenix dactylifera] |
| TRINITY_DN18323_c0_g3 | -1.82 | At3g47200 | UPF0481 protein [Apostasia shenzhenica] |
| TRINITY_DN21808_c0_g1 | -2.94 | BAM3 | PREDICTED: beta-amylase 3, chloroplastic-like [Musa acuminata subsp. malaccensis] |
| TRINITY_DN22422_c0_g1 | 1.79 | WAXY | waxy protein [Aegilops comosa] |
| TRINITY_DN22955_c0_g1 | -2.97 | Pygb | glycosyltransferase family 35 protein [Micromonas commoda] |
| TRINITY_DN2429_c0_g2 | -3.00 | PGIP2 | polygalacturonase inhibitor 1 precursor [Zea mays] |
| TRINITY_DN2716_c0_g3 | 3.41 | AMY1.6 | alpha-amylase isozyme 3D [Dendrobium catenatum] |
| TRINITY_DN2891_c0_g1 | -1.15 | At2g27500 | glucan endo-1,3-beta-glucosidase 14 [Phoenix dactylifera] |
| TRINITY_DN89056_c0_g1 | -3.62 | BAM3 | beta-amylase PCT-BMYI [Solanum tuberosum] |
| TRINITY_DN89383_c0_g1 | -3.01 | PGIP1 | PREDICTED: polygalacturonase inhibitor-like [Musa acuminata subsp. malaccensis] |
| TRINITY_DN90021_c0_g1 | -2.12 | Ugp2 | PREDICTED: UTP--glucose-1-phosphate uridylyltransferase 2-like [Tarenaya hassleriana] |
| TRINITY_DN90899_c0_g1 | -4.14 | GPI | glucose-6-phosphate isomerase-like [Quercus suber] |
| TRINITY_DN9248_c0_g1 | -3.99 | BAM1 | beta-amylase 1, chloroplastic [Phoenix dactylifera] |
| TRINITY_DN9775_c0_g1 | -1.41 | TPS9 | LOW QUALITY PROTEIN: probable alpha,alpha-trehalose-phosphate synthase [UDP-forming] 9 [Asparagus officinalis] |
| TRINITY_DN47018_c0_g1 | -3.42 | BAM3 | beta-amylase 3, chloroplastic [Phoenix dactylifera] |
| TRINITY_DN100706_c0_g1 | 1.12 | SSII-2 | soluble starch synthase 2-2, chloroplastic/amyloplastic [Asparagus officinalis] |
| TRINITY_DN104316_c0_g1 | -2.08 | BAM1 | beta-amylase 1, chloroplastic [Cinnamomum micranthum f. kanehirae] |
| TRINITY_DN105854_c0_g1 | 2.20 | At3g13560 | glucan endo-1,3-beta-glucosidase 4 isoform X1 [Ananas comosus] |
| TRINITY_DN10938_c0_g1 | -1.46 | At4g20820 | berberine bridge enzyme-like 18 [Phoenix dactylifera] |
| TRINITY_DN10938_c0_g2 | -4.01 | At5g44360 | berberine bridge enzyme-like 22 [Asparagus officinalis] |
| TRINITY_DN112811_c0_g1 | 2.26 | AGPC | AGPS1 [Gladiolus hybrid cultivar] |
| TRINITY_DN116517_c0_g2 | 1.16 | SS1 | soluble starch synthase 1, chloroplastic/amyloplastic isoform X1 [Phoenix dactylifera] |
| TRINITY_DN118082_c0_g1 | 1.84 | GBSS | granule-bound starch synthase chloroplastic/amyloplastic-like [Trifolium pratense] |
| TRINITY_DN1273_c0_g1 | -1.85 | AMY3 | alpha-amylase 3, chloroplastic isoform X1 [Phoenix dactylifera] |
| TRINITY_DN13157_c0_g1 | -3.04 | GLU2 | endoglucanase 10-like [Asparagus officinalis] |
| TRINITY_DN14012_c0_g1 | 2.06 | SUS4 | hypothetical protein B296_00039103 [Ensete ventricosum] |
| TRINITY_DN14955_c0_g1 | -1.58 | SUS1 | SuSy protein [Gladiolus hybrid cultivar] |
| TRINITY_DN15042_c0_g1 | -2.10 | GLC1 | Glucan endo-1,3-beta-glucosidase [Apostasia shenzhenica] |
| TRINITY_DN33144_c0_g1 | -1.75 | GH3.1 | PREDICTED: probable indole-3-acetic acid-amido synthetase GH3.1 [Elaeis guineensis] |

**Supplementary Table 6 List of ABA biosynthesis pathway genes in leaves analysed by KEGG enrichment**

| Gene_ID | log_2_FC | Name | Annotation |
| --- | --- | --- | --- |
| TRINITY_DN3974_c0_g1 | -1.95 | D27 | beta-carotene isomerase D27, chloroplastic isoform X1 [Asparagus officinalis] |
| TRINITY_DN4336_c0_g1 | -1.63 | ZEP | zeaxanthin epoxidase, chloroplastic [Asparagus officinalis] |
| TRINITY_DN44880_c0_g1 | 3.24 | UGT73B5 | scopoletin glucosyltransferase-like isoform X1 [Asparagus officinalis] |
| TRINITY_DN4545_c0_g1 | 1.40 | D27 | beta-carotene isomerase D27, chloroplastic [Asparagus officinalis] |
| TRINITY_DN8094_c0_g1 | -1.04 | CRTISO | PREDICTED: prolycopene isomerase, chloroplastic [Elaeis guineensis] |
| TRINITY_DN16860_c0_g2 | -1.14 | PSY | phytoene synthase, chloroplastic-like [Dendrobium catenatum] |
| TRINITY_DN17913_c0_g1 | 2.91 | TOGT1 | UDP-glucose-dependent glucosyltransferase UGT703C1 [Crocus sativus] |
| TRINITY_DN1873_c0_g1 | -1.31 | VDE1 | PREDICTED: violaxanthin de-epoxidase, chloroplastic isoform X1 [Elaeis guineensis] |
| TRINITY_DN22464_c0_g1 | -1.11 | NCED | plastid 9-cis-epoxycarotenoid dioxygenase [Crocus sativus] |
| TRINITY_DN2378_c0_g1 | 3.37 | hpxO | zeaxanthin epoxidase [Gladiolus x hybridus] |
| TRINITY_DN286_c0_g1 | -2.18 | LOC105034391 | PREDICTED: uncharacterized protein LOC105034391 isoform X1 [Elaeis guineensis] |
| TRINITY_DN4831_c0_g3 | 3.62 | TOGT1 | PREDICTED: scopoletin glucosyltransferase-like [Elaeis guineensis] |
| TRINITY_DN4862_c0_g1 | -1.56 | ZEP | zeaxanthin epoxidase, chloroplastic-like [Ananas comosus] |
| TRINITY_DN10818_c0_g1 | 2.48 | ZCD | chromoplast carotenoid cleavage dioxygenase 4a [Crocus sativus] |
| TRINITY_DN10818_c0_g2 | 1.10 | ZCD | chromoplast carotenoid cleavage dioxygenase 4a [Crocus sativus] |
| TRINITY_DN12511_c0_g1 | -2.22 | GT7 | PREDICTED: scopoletin glucosyltransferase-like [Elaeis guineensis] |
| TRINITY_DN13455_c0_g1 | -1.23 | ZSD1 | zerumbone synthase [Asparagus officinalis] |
| TRINITY_DN13686_c0_g1 | -1.34 | CA2 | chromoplast beta-carotene hydroxylase [Crocus ancyrensis] |
| TRINITY_DN1411_c2_g2 | -1.93 | CCD4 | CCD4c [Crocus sativus] |
