# Supplementary material for: Low light reduces saffron corm yield by inhibiting starch synthesis
Source: Front Plant Sci. 2025 Jan 31;16:1544054. doi: 10.3389/fpls.2025.1544054 (PMC11825349; doi:10.3389/fpls.2025.1544054)
Supplement: Supplementary file 2 [file DataSheet2.zip › Supplemental Figures.doc]

**Supplementary Figure 1 The daily sunshine duration, maximum surface solar radiation, average air temperature, and precipitation from November 2022 to April 2023 (A, C) and from December 2023 to April 2024 (B, D).**

**Supplementary Figure 2 Effect of LL on photosynthesis light-response curves (A) and photosynthetic parameters (B) of leaves.** The experiments conducted in March, 2024. The plants were grown under low light intensity (LL) and normal light intensity (NL). All data were the mean ± standard error of three replicates. Significant differences between LL and NL treatments were determined using Student's t-test, ***p*<0.01, ****p*<0.001. LCP, Light compensation point. LSP, Light saturation point. P_nmax,_ Maximum net photosynthetic rate. R_d_, Dark respiration rate. α, Initial quantum efficiency.

**Supplementary Figure 3 Effect of LL on chlorophyll fluorescence parameters of leaves.** The experiments conducted in March, 2024. The plants were grown under low light intensity (LL) and normal light intensity (NL). All data were the mean ± standard error of three replicates. Significant differences between LL and NL treatments were determined using Student's t-test, ***p*<0.01. Y(Ⅱ), Actual photochemical efficiency. ETR, Relative electron transfer rate of PS II. qP, Photochemical quenching coefficient. Fv/Fm, Maximal photochemical efficiency. NPQ, Non-photochemical quenching coefficient.

**Supplementary Figure 4 Effect of LL on the concentration of sucrose (mg g^-1^) (A), glucose (mg g^-1^) (B) and starch (mg g^-1^) (C) in the mother corm.** The experiments conducted in 2023~2024, and the plants were grown under low light intensity (LL) and normal light intensity (NL). Samples were manipulated monthly from January to March in 2024. All data were the mean of three replicates. Significant differences between LL and NL treatments were determined using Student's t-test: **p*<0.05, ***p*<0.01.

**Supplementary Figure 5 Effect of LL on the concentration of sucrose (mg g^-1^) (A) and glucose (mg g^-1^) (B) in leaves.** The experiments conducted in 2023~2024, and the plants were grown under low light intensity (LL) and normal light intensity (NL). Samples were manipulated monthly from January to March in 2024. All data were the mean of three replicates. Significant differences between LL and NL treatments were determined using Student's t-test: **p*<0.05, ***p*<0.01.

**Supplementary Figure 6 Effect of LL on the concentration of sucrose (A), glucose (B) and starch (C) in daughter corms.** The experiments conducted in 2023~2024, and the plants were grown under low light intensity (LL) and normal light intensity (NL). Samples were manipulated monthly from February to March in 2024. All data were the mean of three replicates. Significant differences between LL and NL treatments were determined using Student's t-test: **p*<0.05, ***p*<0.01.

**Supplementary Figure 7 Analysis of differential gene expression in leaves and daughter corms.** The experiments conducted in 2022~2023, and the plants were grown under low light intensity (LL) and normal light intensity (NL). Samples were manipulated in March, 2023. All data were the mean of three replicates. The screening criteria for DEGs is, |log_2_fold change (FC)| ≥1, *p*-value <0.05. A, The number of the differential expressed genes (DEGs) in different gruops. B, Volcano plot of DEGs in leaves (LLvsNL). C, Volcano plot of DEGs in daughter corms (LLvsNL).

**Supplementary Figure 8 Effect of LL on the expression levels of key genes in the respiratory metabolic pathway of leaves.** The experiments conducted in 2022~2023, and the plants were grown under low light intensity (LL) and normal light intensity (NL). Samples were manipulated in March of the following year. The expression levels of key genes involved in the glycolysis, pyruvate metabolism and tricarboxylic acid cycle pathways in the transcriptome were represented by heat maps. Compared to NL, the Log_2_FC value was used as the relative expression level of genes in LL leaves. F-6-P, Fructose-6-phosphate. F-D-P, Fructose-1,6-Diphosphate. G-3-P, 3-glyceraldehyde phosphate. 1,3-D-G-P, 1,3-diphosphoglycerate. 3-PGA, 3-phosphoglycerate. 2-PGA, 2-Phosphoglycerate. PEP, phosphoenolpyruvate. Rectangle represents *p*>0.05 in differential gene analysis, while circle represents *p*< 0.05.

**Supplementary Figure 9 Effect of LL on gene expression of ABA biosynthesis in daughter corms.** The experiments conducted in 2022~2023, and the plants were grown under low light intensity (LL) and normal light intensity (NL). Samples were manipulated in March of the following year. All data were the mean of three replicates. The expression levels of the genes have been normalized to TPM values.
